# Isoflavone Malonyltransferases GmIMaT1 and GmIMaT3 Differently Modify Isoflavone Glucosides in Soybean (*Glycine max*) under Various Stresses

**DOI:** 10.3389/fpls.2017.00735

**Published:** 2017-05-16

**Authors:** Muhammad Z. Ahmad, Penghui Li, Junjie Wang, Naveed Ur Rehman, Jian Zhao

**Affiliations:** National Key Laboratory of Crop Genetic Improvement, Huazhong Agricultural UniversityWuhan, China

**Keywords:** BAHD family, malonyltransferase, isoflavone malonate, enzyme kinetics, stress response

## Abstract

Malonylated isoflavones are the major forms of isoflavonoids in soybean plants, the genes responsible for their biosyntheses are not well understood, nor their physiological functions. Here we report a new benzylalcohol *O*-acetyltransferase, anthocyanin *O*-hydroxycinnamoyltransferase, anthranilate N-hydroxycinnamoyl/benzoyltransferase, deacetylvindoline 4-*O*-acetyltransferase (BAHD) family isoflavone glucoside malonyltransferase GmIMaT1, and GmIMaT3, which is allelic to the previously characterized GmMT7 and GmIF7MaT. Biochemical studies showed that recombinant GmIMaT1 and GmIMaT3 enzymes used malonyl-CoA and several isoflavone 7-*O*-glucosides as substrates. The *K*_m_ values of GmIMaT1 for glycitin, genistin, and daidzin were 13.11, 23.04, and 36.28 μM, respectively, while these of GmIMaT3 were 12.94, 26.67, and 30.12 μM, respectively. Transgenic hairy roots overexpressing both *GmIMaT*s had increased levels of malonyldaidzin and malonylgenistin, and contents of daidzin and glycitin increased only in *GmIMaT1*-overexpression lines. The increased daidzein and genistein contents were detected only in *GmIMaT3*-overexpression lines. Knockdown of *GmIMaT1* and *GmIMaT3* reduced malonyldaidzin and malonylgenistin contents, and affected other isoflavonoids differently. GmIMaT1 is primarily localized to the endoplasmic reticulum while GmIMaT3 is primarily in the cytosol. By examining their transcript changes corresponding to the altered isoflavone metabolic profiles under various environmental and hormonal stresses, we probed the possible functions of GmIMaTs. Two *GmIMaT*s displayed distinct tissue expression patterns and respond differently to various factors in modifying isoflavone 7-*O*-glucosides under various stresses.

## Introduction

**FIGURE 1:**
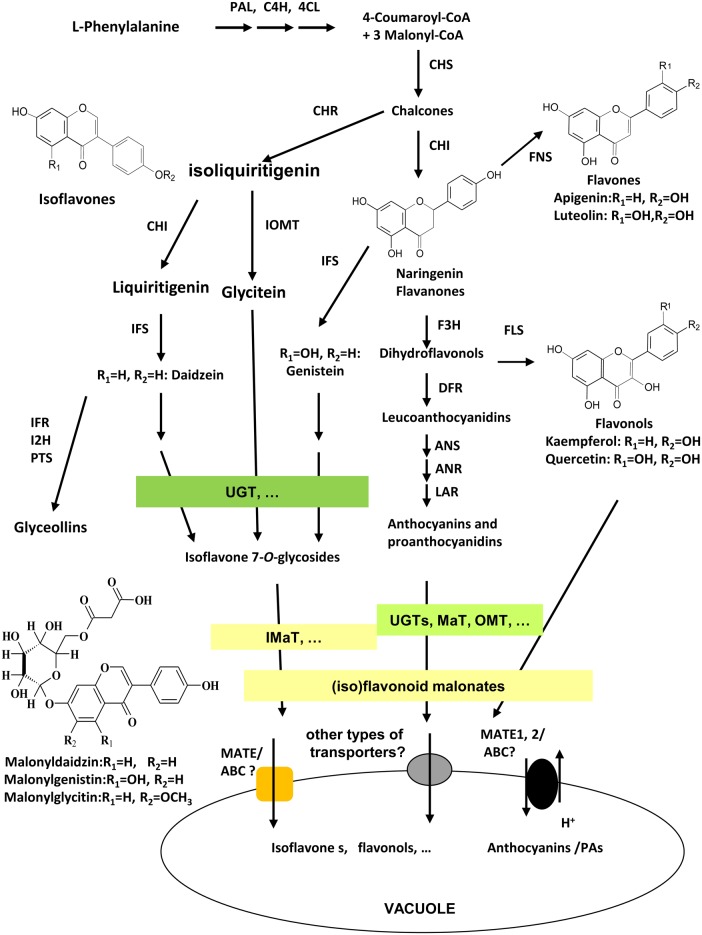
**Scheme of (iso)flavonoid biosynthesis, modification, and transport in soybean**. Isoflavonoid biosynthesis starts from legume-specific branches at IFS/CHI-catalyzed steps from phenylpropanoid pathway. Flavonoids and isoflavonoids share naringenin chalcones as common precursors. Enzymes involved in the biosynthesis pathway as following; PAL, phenylalanine ammonia-lyase; C4H, cinnamate 4-hydroxylase; 4CL, 4-coumarate CoA ligase; CHS, chalcone synthase; CHI, chalcone isomerase; CHR, chalcone reductase; IFS, isoflavone synthase; I2′H, isoflavone 2′-hydroxylase; IFR, isoflavone reductase; IOMT, isoflavonoid *O*-methyltransferase; HID, 2-hydroxyisoflavanone dehydratase; F3H, flavanone 3-hydroxylase; FLS, flavonol synthase; FNS, flavone synthase; DFR, dihydroflavonol 4-reductase; ANS, anthocyanidin synthase; ANR, anthocyanidin reductase; UGT, UDP-glucose:flavonoid glycosyltransferase; MaT, Malonyl-CoA: flavonoid glucoside acyltransferase; MATE1/TT12, multidrug and toxic extrusion protein 1/transparent testa 12; ABC, ATP-binding cassette; PTS, pterocarpan synthases.

As unique to legume plants, isoflavonoids co-exist with flavonoids in many tissues and organs in legumes with various biological functions. Isoflavonoids play a vital role in plant defense response against different biotic and abiotic stresses, such as plant-pathogen by acting as precursors for phytoalexins biosynthesis (Graham and Graham, 1991; Dixon, 2001; Zhao et al., 2005;Akashi et al., 2009; Zhou et al., 2011; Ishiga et al., 2015; Yoneyama et al., 2016), plant-insects (Murakami et al., 2014; Zavala et al., 2015; Nakata et al., 2016), plant-symbionts by providing signals for Nod gene stimulation and nodule development (Subramanian et al., 2007; Zhang et al., 2009; Yasuda et al., 2016), and abiotic environmental stresses (Gutierrez-Gonzalez et al., 2010; Tripathi et al., 2016). They also contribute significantly to health beneficiary properties of legume seed foods for improving human health against heart disease, female breast cancer, and cardiovascular disease (Shin and Lee, 2013; Lee et al., 2016). Among numerous (iso)flavonoids in legumes, a large number of (iso)flavonoids are modified into malonates, that accumulate in various tissues and different amounts of *Medicago truncatula* (Farag et al., 2008; Yu et al., 2008; Zhao et al., 2011). The major isoflavonoids in soybean plants are daidzein, genistein, and glycitein aglycones and their glucoside and malonate derivatives. The biosynthesis of isoflavonoids through phenylpropanoid pathway have been extensively studied, and genes involved in early steps have been characterized clearly, yet genes involved in late steps, particularly, in extensive modifications, transport, and storage of isoflavones, as well as the complex mechanisms underlying the regulation of isoflavone biosynthesis, are not fully understood (**Figure 1**; Zhao, 2015). The 3-, 5-, 7-, or 3′-OH groups of glycosylation, further acylation, methylation, and hydroxylation modification in isoflavonoids generate diverse groups of compounds (**Figure 1**; Bowles et al., 2005; D’Auria et al., 2007; Ferrer et al., 2008). Daidzein 7-*O*-glucoside, genistein 7-*O*-glucoside, and glycitein 7-*O*-glucoside, and their malonylated forms like 6′′-*O*-malonyldaidzin, 6′′-*O*-malonylgenistin, and 6′′-*O*-malonylglycitin are the major components in Medicago and soybean tissues (Kudou et al., 1991; Suzuki et al., 2007; Yu et al., 2008). The Uridine diphosphate-dependent glycosyltransferase (UGTs) and malonyl CoA-dependent acyltranferases (MATs) catalyze successive steps of modification of isoflavone aglycones in soybean (Dhaubhadel et al., 2008; Funaki et al., 2015).

Glycosylation and malonylation are important modifications of (iso)flavonoids, conferring the solubility, stability, and transport properties for storage into the vacuole (**Figure 1**; Luo et al., 2007; Zhao et al., 2011; Funaki et al., 2015; Rinaldo et al., 2015). The benzylalcohol *O*-acetyltransferase, anthocyanin *O*-hydroxycinnamoyltransferase, anthranilate N-hydroxycinnamoyl/benzoyltransferase, deacetylvindoline 4-*O*-acetyltransferase (BAHD) family acyltransferases are identified as enzymes catalyzing acyl-CoA (including acetyl-CoA, malonyl-CoA, coumaroyl-CoA, etc.)-dependent modification of (iso)flavonoids, including anthocyanins, flavonols glucosides, and isoflavone glucosides (D’Auria et al., 2007; Luo et al., 2007; Yu et al., 2008; Zhao et al., 2011). The extensive malonylation of isoflavone glucosides may have physiological functions yet to be identified, although the malonylation may stabilize (iso)flavonoids, enhance their solubility, or facilitate their transport or storage in Arabidopsis and Medicago (Luo et al., 2007; Yu et al., 2008). Previous studies have demonstrated that malonylation of flavonoids are essential for them to be chemically more stable and readily transported into the vacuole by multidrug and toxin extrusion (MATE) transporter (Zhao et al., 2011). In yeast elicitor or methyl jasmonate (MeJA)-treated Medicago cell cultures, many isoflavone malonates were stored in the vacuole for the purpose of defense (Naoumkina et al., 2007; Zhao, 2015). Because the functions of isoflavonoids may depend on the existence or lack of conjugation, the isoflavonoid malonates may also act as intermediates to direct the symbiosis interaction between legume and rhizobia (Funaki et al., 2015). Legumes like soybean and Medicago genome contain a number of MaT homologous (Yu et al., 2008; Schmutz et al., 2010). Up to date, only MtMaT1, MtMaT4, MtMaT5, and MtMaT6 were characterized from *M. truncatula* (Yu et al., 2008; Zhao et al., 2011), and GmMT7 and GmIF7MaT are the only characterized isoflavone 7-*O*-glucoside malonyltransferases from soybean seeds with only one amino acid difference at W240R (Suzuki et al., 2007; Dhaubhadel et al., 2008). Both proteins use malonyl-CoA as the only acyl donor to convert daidzin, genistin, and glycitin into the corresponding daidzin, genistin, and glycitin 6′′-*O*-malonates (Suzuki et al., 2007; Dhaubhadel et al., 2008).

In an attempt to understand why and how soybean plants generate so many different isoflavone glucoside malonates in various amounts, we found many redundant flavonoid malonyltransferase genes in soybean genome and tried to characterize some of them. We here report one new soybean BAHD family malonyltransferase GmIMaT1 and a new allele of GmIF7MaT/GmMT7, GmIMaT3 with clear biochemical properties and physiological functions. GmIMaT3 has three amino acid differences from GmMT7 and GmIF7MaT (**Supplementary Figure S1B**; Suzuki et al., 2007; Dhaubhadel et al., 2008). Both GmIMaT1 and GmIMaT3 recombinant proteins can convert isoflavone 7-*O*-glucosides into their corresponding 6′′*-O*-malonates isoflavonoids. Their overexpression or knockdown transgenic soybean hairy roots displayed the altered isoflavonoid profiles. In soybean plant, both GmIMaTs showed very different gene expression patterns, not only in tissue-specific manners, but also in response to various stresses and hormone regulators. The changes of their expression levels in soybean plants did not often coincide with the alteration in isoflavone profiles, in particular, the malonylisoflavonoids. Our data suggest that while GmIMaT1 and GmIMaT3 modification of isoflavones may play important roles in plant growth and adaptations to various environmental or hormonal stresses, other redundant isoflavone malonyltransferases also function in complementary. These may enhance the flexibility of soybean isoflavones in needs for growth and development and adaptation to environmental changes.

## Results

### Identification of Two Isoflavone Malonyltransferases from Soybean

**FIGURE 2:**
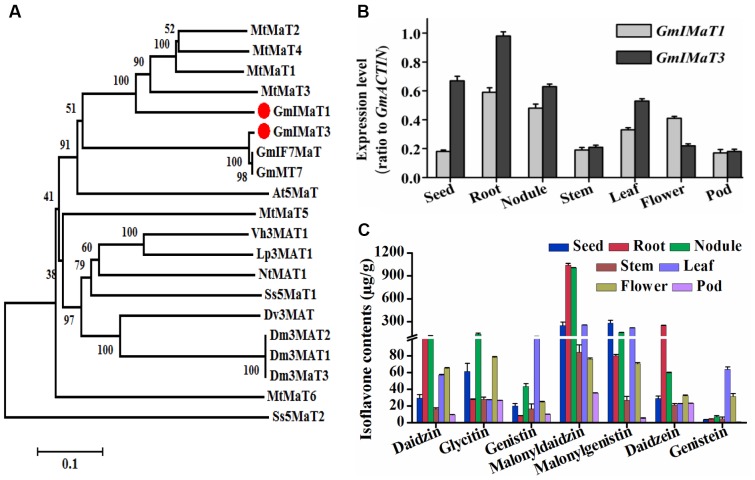
**Identification of GmIMaT1 and GmIMaT3 with expression and isoflavone profiles in soybean tissues. (A)** Phylogenetic analysis of GmIMaT1 and GmIMaT3 with other benzylalcohol *O*-acetyltransferase, anthocyanin *O*-hydroxycinnamoyltransferase, anthranilate N-hydroxycinnamoyl/benzoyltransferase, deacetylvindoline 4-*O*-acetyltransferase (BAHD) family acyltransferases. Rooted phylogenetic tree for BAHD malonyltransferases was constructed by using MEGA6 program through neighbor joining method. Bar shows 0.1 amino acid substitution site. **(B)** Expression patterns of *GmIMaT1* and *GmIMaT3* in different soybean tissues. Seeds, nodules, and flower from 8 to 11 weeks old plants, root, stem, and leaf from 12 to 15 days old seedling were harvested for quantitative reverse transcription (qRT-PCR) polymerase chain reaction. Transcript levels are expressed relative to that of *GmACTIN* (Glyma19G147900.1). **(C)** Isoflavone contents in different tissues of soybean plant. Flavonoids were extracted with 80% methanol and analyzed with high pressure liquid chromatography (HPLC); the isoflavone contents were calculated by standard curves. Data are expressed in means ± SD from at least three independent experiments with duplicates.

Soybean plant produces three major types of isoflavones, daidzein, genistein, and glycitein aglycones and their glucosides and malonylglucosides. Among them, the malonylglucosides are the most abundant forms in soybean most tissues (Li et al., 2016). To characterize the genes responsible for generation of isoflavone malonylglucosides, we used the characterized GmMT7 and Medicago MATs as baits to search soybean genome. Among more than 100 BAHD family genes from soybean, we cloned several most closest homologs, including Glyma18G50320 (representing its tandem duplicated genes Glyma18G50350, Glyma18G50310, and Glyma18G50330), Glyma18G49240, Glyma13G06230, Glyma19G03770, and Glyma18G50340. However, only some of them are functionally active, while others, such as Glyma18G49240 and Glyma18G50340, did not show any activity when using recombinant proteins under our assay conditions. Two malonyltransferase genes GmIMaT1 (Glyma18G50320, v1.0 or Glyma.18G268200.1, v2.0) and GmIMaT3 (Glyma13G06230, v1.0 or Glyma.13G056100.1, v2.0) were characterized and reported for their functions here. *GmIMaT1* and *GmIMaT3* encode peptides of 476 and 467 amino acids, respectively. The HXXXD and DFGWG motifs conservatively present in GmIMaT1 and GmIMaT3 protein sequences, indicating that they belong to BAHD acyltransferase superfamily (St-Pierre and De Luca, 2000). The anthocyanin specific motif 2 (YFGNC) is also found between motif 1 and motif 3 (**Supplementary Figure S1A**). GmIMaT1 shares 62% identity and 76% similarity with MtMaT2, followed by 58% identity, 73% similarity with MtMaT4, while GmIMT3 shares 99% identity and similarity with its allele GmMT7 and IF7MaT, 43% identity with MtMaT1 and 59% similarity with MtMaT1 and MtMaT2 (**Supplementary Table S2**, Supplementary Notes 1 and 2). GmIMaT1 and GmIMaT3 are clustered with other characterized isoflavone malonyltransferases GmMT7, MtMaT1, MtMaT2, MtMaT3, and MtMaT4 in a phylogenetic analysis (**Figure 2A**; Yu et al., 2008; Zhao et al., 2011).

We examined their tissue expression patterns with quantitative reverse transcription (qRT-PCR) polymerase chain reaction. The higher transcript levels of both *GmIMaT1* and *GmIMaT3* were detected in roots and nodules, but very low in stem and pod. The transcript level of *GmIMaT1* was highest in roots and nodule, then in leaf and flower, suggesting that GmIMaT1 may play a specific role in the malonylation of isoflavone glucosides in root and nodule. However, *GmIMaT3* transcripts were the highest in root, and then in seed and nodule, followed by leaf (**Figure 2B**). These expression patterns for two *GmIMaT*s were basically similar to public databases (**Supplementary Figure S2**). To understand the different physiological roles of GmIMaT1 and GmIMaT3, we analyzed isoflavones in these tissues (**Figure 2C**). Comparison of *GmIMaT1* and *GmIMaT3* transcript levels in different tissues with isoflavone contents shows that different tissues produce distinct types of isoflavones, daidzein, glycitein, and genistein aglycones, or in forms of glucosides and glucoside malonates (**Figure 2C**). However, the tissues with higher *GmIMaT1* and *GmIMaT3* transcript levels, such as root, seed, nodule, and leaf, generally accumulate higher malonyl isoflavones, suggesting these two GmIMaT may account for the production of the major portions of isoflavone glucoside malonates.

### *GmIMaTs* Showed Isoflavone Malonyltransferase Activity toward Multiple Substrates

**FIGURE 3:**
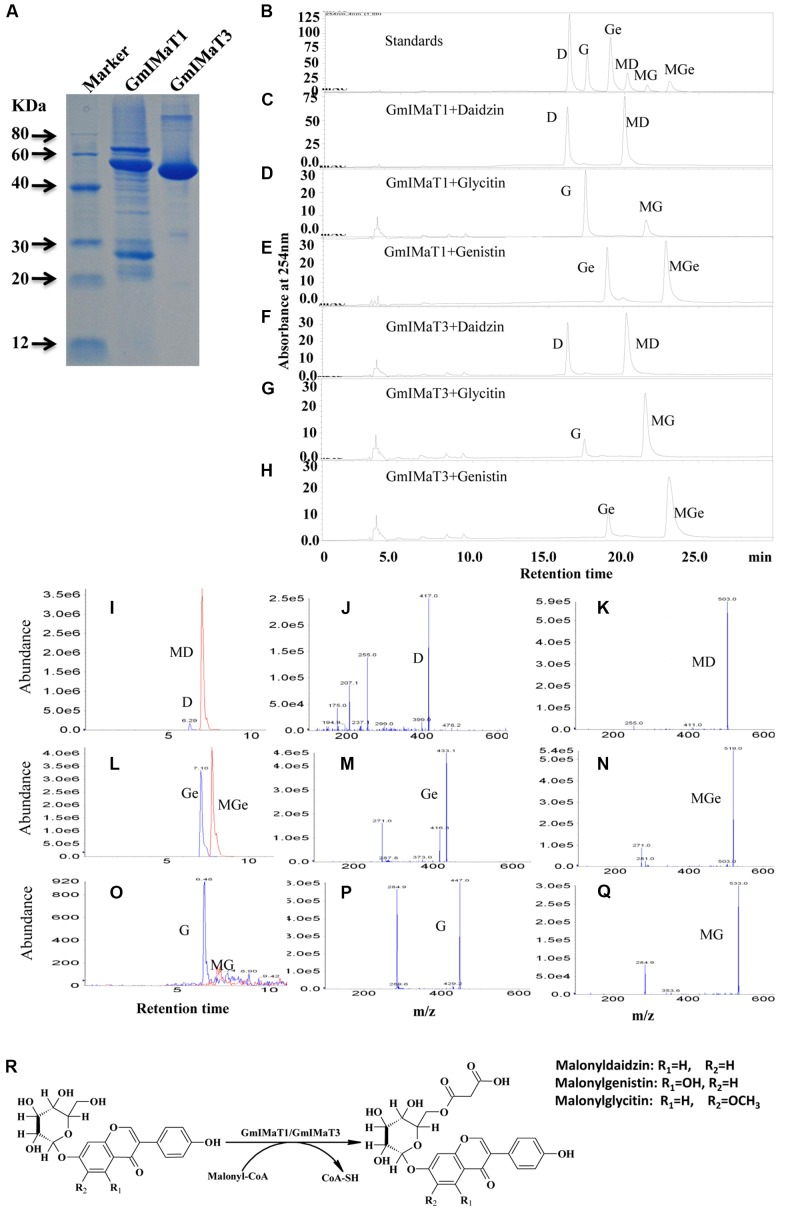
**GmIMaT1 and GmIMaT3 activity assay. (A)** The purified recombinant GmIMaT1 and GmIMaT3. *GmIMaT1* and *GmIMaT3* were expressed in *E. coli* in His-tagged fusions and partially purified with nickel resin **(A)**. Proteins were resolved on sodium dodecyl sulfate–polyacrylamide gel electrophoresis (SDS-PAGE), followed by staining with Coomassie Blue R-250. **(B–H)** GmIMTa1 and GmIMaT3 activity assay. The recombinant proteins were incubated in malonyltransferase reaction with various isoflavone glucosides and malonyl-CoA as substrates. The enzymatic products were analyzed with HPLC as compared with isoflavonoid standards **(B)**, reaction analysis for recombinant GmIMaT1 with daidzin **(C)**, glycitin **(D)**, genistin **(E)** as substrates. The reaction analysis for recombinant GmIMaT3 with daidzin **(F)**, glycitin **(G)**, genistin **(H)** as substrates. The symbols are D, daidzin; G, glycitin; Ge, genistin; MD, malonyldaidzin; MG, malonylglycitin; MGe, malonylgenistin. **(I–R)** Verification of malonylation products from corresponding substrates with mass spectrometry for recombinant GmIMaT1 and GmIMaT3. Mass spectra for daidzin and malonyldaidzin **(I)**, genistin and malonylgenistin **(L)**, glycitin and malonylglycitin **(O)** in GmIMaT1-catalyzed reactions. Mass spectra for glucoside daidzin **(J)**, genistin **(M)**, and glycitin **(P)**. Mass spectra for malonyldaidzin **(K)**, malonylgenistin **(N)**, and malonylglycitin **(Q)**. D, daidzin; G, glycitin; Ge, genistin; MD, malonyldaidzin; MG, malonylglycitin; MGe, malonylgenistin. **(R)** The malonylation of isoflavone 7-*O*-glucosides (daidzin, genistin, and glycitin) at 6′′ position catalyzed by malonyltransferases (GmIMaT1/GmIMaT3) to generate malonylglucosides (malonyldaidzin, malonylgenistin, and malonylglycitin).

We expressed *GmIMaT1* and *GmIMaT3* in His-tagged fusion protein in *E. coli* strain BL21 (DE3) and purified with nickel-resin (**Figure 3A**). Both recombinant enzymes gave the maximum activity at pH 7.2 when we used malonyl CoA as acyl-donor and genistin as an acceptor (**Supplementary Figure S3**). With these partially purified recombinant enzymes, we determined acyltransferase activity by using malonyl CoA and acetyl CoA as donors and three isoflavone glucosides, daidzin, glycitin, and genistin, and flavonoid glucosides (cyanidin-3-*O*-glucoside, myricitrin, naringin 7-*O*-glucoside, quercitrin, vitexin, and rutin) as acceptor substrates. We found that both GmIMaT1 and GmIMaT3 preferred to use malonyl-CoA, rather than acetyl CoA, as an acyl donor to convert only isoflavone 7-*O*-glucosides, but not other flavonoid glucosides, into corresponding malonylglucosides (**Figures 3B–H**). The enzymatic reactions were analyzed with both high pressure liquid chromatography (HPLC) and liquid chromatography-coupled tandem mass spectrometry (LC-MS/MS) to confirm the reaction product with authentic standards. GmIMaT1 and GmIMaT3 enzymes converted isoflavone 7-*O*-glucosides, daidzin into malonyldaidzin, genistin into malonylgenistin, and glycitin into malonylglycitin, as confirmed by LC-MS/MS (**Figures 3I–Q**). The LC-MS/MS profiles showed the same peaks and identical spectrum to the products of isoflavone 7-*O*-(6′′-*O*-malonyl)-β-D-glucosides on 6′′-position isoflavone sugar as reported in GmMT7 (Dhaubhadel et al., 2008) and *M. truncatula* MaT1, MaT2, MaT3, and MaT4, with the increase malonyl group mass of 86 *m/z* as compared with substrates (**Figures 3I–Q**; Yu et al., 2008; Zhao et al., 2011).

Lineweaver–Burk plot was generated to analyze the kinetics of these enzymes, such as the apparent *K*_m_ and *V*_max_ values (**Supplementary Figure S4**). Both enzymes showed same trends toward their substrate specificity but the values of kinetic parameters were quite different. Both showed maximum *K*_m_ value for daidzin, followed by genistin and glycitin while vice versa for *V*_max_ at the saturated concentrations of substrates. GmIMaT1 showed the largest *K*_cat_/*K*_m_ 5.21 S^-1^ μM^-1^ for glycitin followed by genistin 2.47 S^-1^ μM^-1^ and minimum for daidzin, suggesting that GmIMaT1 has the highest binding affinity for glycitin and genistin. This is similar to GmIMaT3, which showed the highest *K*_cat_/*K*_m_ value for glycitin (5.61 S^-1^ μM^-1^), genistin (1.54 S^-1^ μM^-1^), and daidzin (1.15 S^-1^ μM^-1^). The *K*_cat_/*K*_m_ values of GmIMaT1 and GmIMaT3 for malonyl CoA were (0.44 S^-1^ μM^-1^) and (0.75 S^-1^μM^-1^), respectively (**Table 1**), when genistin glucoside was used as acyl-acceptor substrate. Therefore, glycitein seemed to be the favorite substrate for both GmIMaTs.

**Table 1:** Kinetic parameters of recombinant GmIMaT1 and GmIMaT3 with substrates.

Substrates	Enzymes	*K*_m_ (μM)	*V*_max_ (nmol mg^-1^ min^-1^)	*K*_cat_ (S^-1^)	*K*_cat_*/K*_m_ (S^-1^ μM^-1^)
Daidzein 7-*O*-glucoside	GmIMaT1	36.28 ± 3.2	148 ± 19	29.63 ± 3.7	0.82 ± 0.01
	GmIMaT3	30.12 ± 2.6	173 ± 27	34.24 ± 2.4	1.15 ± 0.12
Genistein 7-*O*-glucoside	GmIMaT1	23.04 ± 1.5	282 ± 13	56.35 ± 2.6	2.47 ± 0.10
	GmIMaT3	26.67 ± 0.86	205 ± 11	40.95 ± 2.3	1.54 ± 0.14
Glycitein 7-*O*-glucoside	GmIMaT1	13.11 ± 0.92	342 ± 27	68.39 ± 5.7	5.21 ± 0.14
	GmIMaT3	12.94 ± 1.3	333 ± 47	66.65 ± 9.3	5.61 ± 1.25
Cyanidin-3-*O*-glucoside	GmIMaT1	nd	nd	nd	nd
	GmIMaT3	nd	nd	nd	nd
Myricitrin	GmIMaT1	nd	nd	nd	nd
	GmIMaT3	nd	nd	nd	nd
Naringin 7-*O*-glucoside	GmIMaT1	nd	nd	nd	nd
	GmIMaT3	nd	nd	nd	nd
Quercitrin	GmIMaT1	nd	nd	nd	nd
	GmIMaT3	nd	nd	nd	nd
Vitexin	GmIMaT1	nd	nd	nd	nd
	GmIMaT3	nd	nd	nd	nd
Rutin	GmIMaT1	nd	nd	nd	nd
	GmIMaT3	nd	nd	nd	nd
Malonyl-CoA for Genistin	GmIMaT1	18.30 ± 1.2	68 ± 4.8	8.12 ± 1.7	0.44 ± 0.07
	GmIMaT3	25.59 ± 2.2	128 ± 14	19.21 ± 1.9	0.75 ± 0.12
Acetyl-CoA	GmIMaT1	nd	nd	nd	nd
	GmIMaT3	nd	nd	nd	nd

*nd, not detected*.

### Overexpression and Silencing of *GmIMaTs* Altered Isoflavone Profiles in Hairy Roots

**FIGURE 4:**
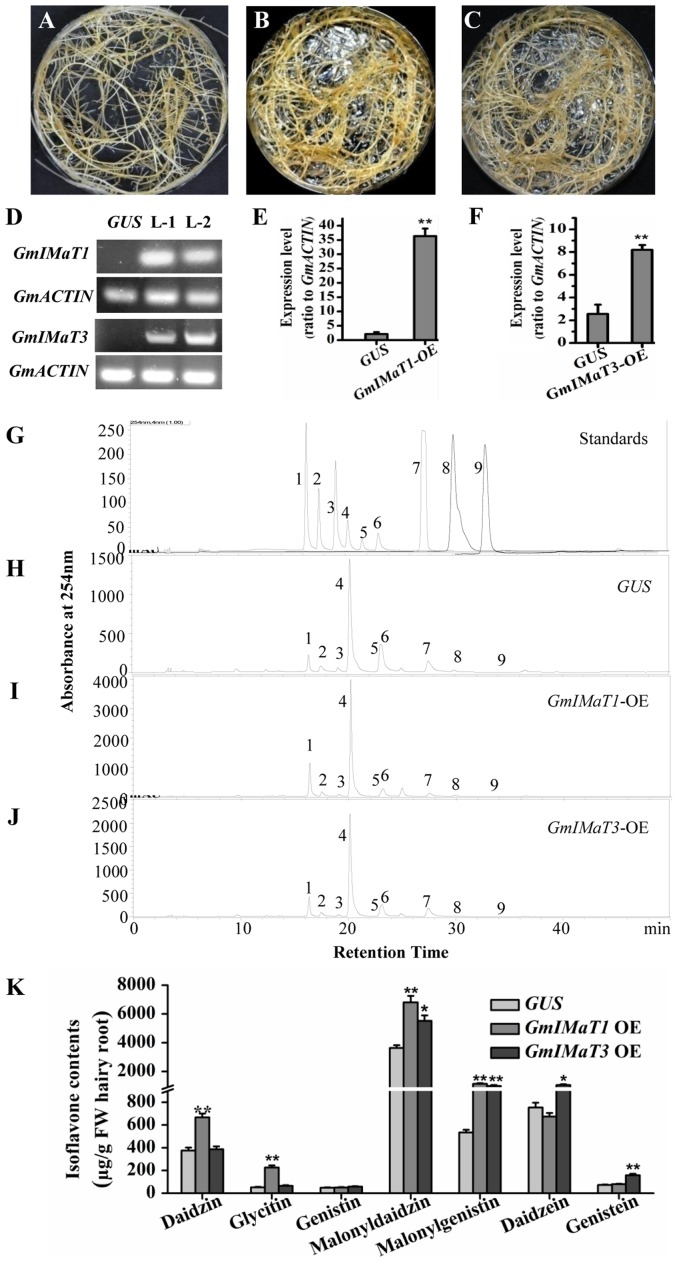
**GmIMaT1 and GmIMaT3 overexpression affects isoflavone contents in hairy roots. (A–C)** Representative hairy roots expressing *GUS* **(A)**, *GmIMaT1*-OE **(B)**, *GmIMaT3*-OE **(C). (D)** Semi-quantitative RT-PCR examination of overexpression of *GmIMaT1-OE* and *GmIMaT3-OE* in representative transgenic hairy root lines. Soybean *GmACTIN* was used as an internal control. L-1, transgenic hairy root line-1; L-2, transgenic hairy root line-2. **(E,F)** qRT-PCR confirmation of *GmIMaT1* and *GmIMaT3* expression in transgenic hairy root lines. **(G–J)** HPLC chromatograms for isoflavone profiles in *GmIMaT1* and *GmIMaT3* overexpression hairy root lines. The numbered peaks are: 1, daidzin; 2, glycitin; 3, genistin; 4, malonyldaidzin; 5, malonylglycitin 6, malonyl genistin; 7, daidzein; 8, glycitein; 9, genistein. **(K)** The contents of isoflavone compounds in *GmIMaT* overexpressed hairy root lines. Data are expressed in means ± SD from at least three independent transgenic lines. Differences between control and *GmIMaT* transgenic lines were analyzed by using Student’s *t*-test in two-tailed comparison. ^∗^*P* < 0.05 and ^∗∗^*P* < 0.01.

We then verified their functions in transgenic *in vitro Glycine max* hairy roots with either overexpression or knockdown of *GmIMaT1* and *GmIMaT3*. The morphologies of transgenic hairy roots overexpressing β-glucuronidase (GUS), GmIMaT1, or GmIMaT3 appeared no difference (**Figures 4A–C**). The expression of *GmIMaT1* and *GmIMaT3* in their transgenic hairy roots were confirmed with both semi qRT-PCR (**Figure 4D**) and qRT-PCR (**Figures 4E,F**). Analysis of isoflavone metabolites in these transgenic hairy roots suggested that daidzin, glycitin, malonyldaidzin, and malonylgenistin contents were significantly increased in *GmIMaT1*-overexpression hairy root lines as compared with *GUS* control (*P* < 0.01). *GmIMaT1-OE* transgenic hairy roots had 2.1-fold increase in malonyldaidzin, 1.5-fold increase in malonylgenistin, compared with *GUS* control lines (**Figures 4H–J**). The *GmIMaT3*-overexpression hairy root lines showed significantly increased contents of isoflavone aglycones and isoflavone glucoside malonylates (*P* < 0.01). *GmIMaT3*-overexpression lines had 50% increase in malonyldaidzin, onefold increase in malonylgenistin, and 80% increase in genistein aglycone contents as compared with *GUS* control. The increase in isoflavone glucosides was not significantly observed in *GmIMaT3*-overexpression lines (**Figure 4K**).

**FIGURE 5:**
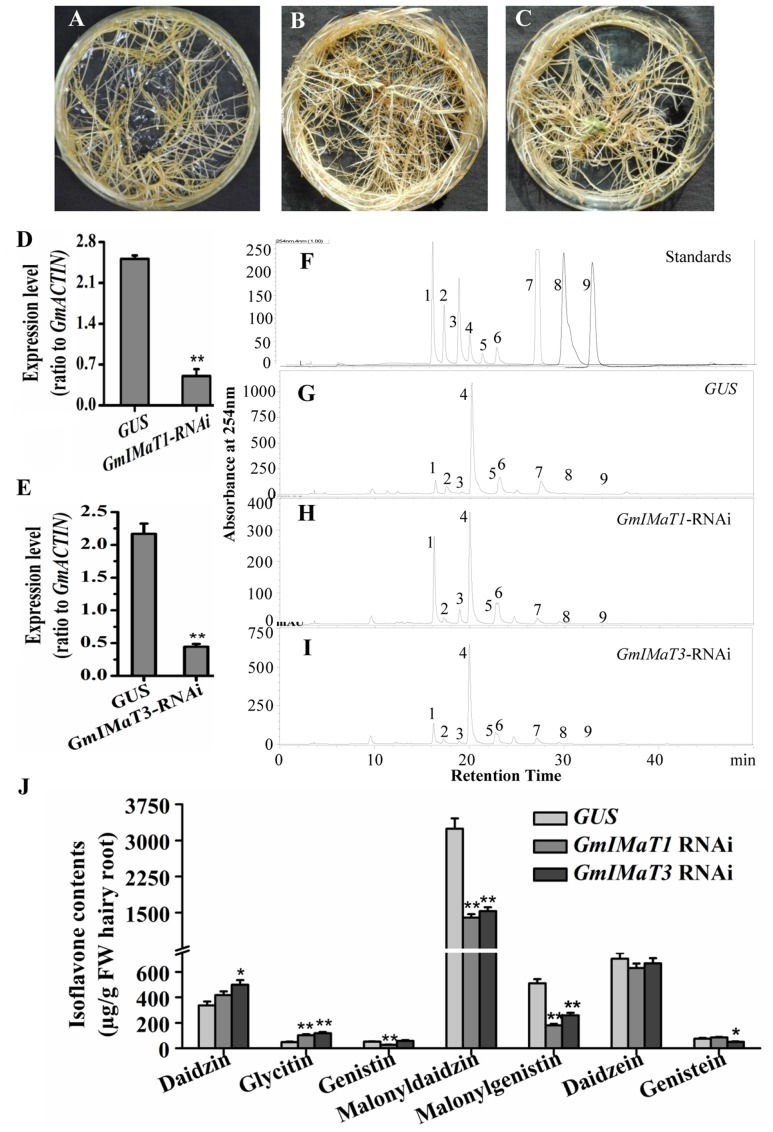
**Effects of *GmIMaT1* and *GmIMaT3* knockdown on isoflavone contents in hairy roots. (A–C)** Representative hairy roots expressing *GUS* **(A)**, *GmIMaT1-RNAi* **(B)**, *GmIMaT3-RNAi* **(C). (D,E)** Expression of *GmIMaT1* **(D)** and *GmIMaT3* **(E)** in transgenic hairy root lines. **(F–I)** HPLC chromatographs for isoflavone profiles in *GmIMaT1* and *GmIMaT3* knockdown hairy root lines. **(F)** The numbered standard peaks are: 1, daidzin; 2, glycitin; 3, genistin; 4, malonyldaidzin; 5, malonylglycitin; 6, malonyl genistin; 7, daidzein; 8, glycitein; 9, genistein. **(J)** Contents of isoflavones in *GmIMaT* knockdown hairy root lines. Data are expressed in means ± SD from at least three independent transgenic lines. Differences between control and *GmIMaT* transgenic lines were analyzed by using Student’s *t*-test in two-tailed comparison. ^∗^*P* < 0.05 and ^∗∗^*P* < 0.01.

The GmIMaT1- and GmIMaT3-silenced transgenic hairy roots were also generated by RNAi method, with no dramatic morphological change as compared with GUS control hairy roots (**Figures 5A–C**), as confirmed by qRT-PCR (**Figures 5D,E**): *GmIMaT1* transcripts remained about 30% and *GmIMaT3* transcripts remained 26% in silenced hairy roots as compared with *GUS* control. As compared with isoflavone standards in soybean hairy roots (**Figure 5F**), *GmIMaT1*- and *GmIMaT3*-silenced hairy roots synthesized significantly fewer isoflavones than the *GUS* control (**Figures 5G–I**). *GmIMaT1*-knockdown hair roots showed about 40% decrease in genistin, 50% decrease in malonyldaidzin and about 80% decrease in malonylgenistin contents as compared to *GUS* control. The other glucosides and aglycone contents were not significantly decreased but increased (**Figure 5J**). The malonyldaidzin, malonylgenistin, and genistein aglycone contents significantly decreased in *GmIMaT3*-RNAi hairy root lines, by about 50, 60, and 25% reduction, respectively, than *GUS* control (**Figure 5I**).

### Subcellular Localization of GmIMaT1 and GmIMaT3

**FIGURE 6:**
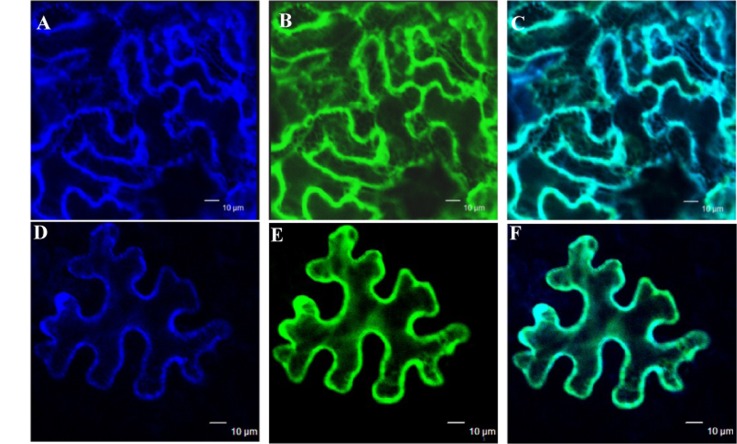
**Subcellular localization of GmIMaT1 and GmIMaT3**. Transient expression of GFP-GmIMaT1, GFP-GmIMaT3 and ER marker CD3-959-mCherry, driven by 35S promoter, was done in epidermal cells of tobacco leaf. Confocal microscopy was used for imaging. Bars = 10 μm. **(A–C)** ER marker CD3-959-mCherry in blue **(A)**, GFP-GmIMaT1 **(B)**, and the merge of both **(C). (D–F)** ER marker CD3-959-mCherry in blue **(D)**, GFP-GmIMaT3 **(E)**, and the merge of both **(F)**.

The subcellular localization of GmIMaT1 and GmIMaT3 was examined to gain further understanding of their biological functions. When GFP-GmIMaT1 and GmIMaT3 fusions were transiently expressed in the epidermal cells of *Nicotiana benthamiana* leaves, GFP-GmIMaT1 signals were largely co-localized with a endoplasmic reticulum (ER) marker CD3-959-mCherry (**Figures 6A–C**). The many network-like GFP-GmIMaT1 signals showed a large degree of overlapping patterns with the ER markers, suggesting that GFP-GmIMaT1 is mainly an ER-localized malonyltransferase (**Figure 6**). GFP-GmIMaT3 did not show clear ER-net-like signals, and no complete overlapping pattern with the ER marker as GFP-GmIMaT1 did. Thus, GFP-GmIMaT3 signals were primarily in the cytosol. It is more likely a cytosol-localized malonyltransferase, perhaps retaining minor signals in the ER (**Figures 6D–F**). This cytosolic localization of GmIMaT3 is consistent with the previous observation on its allele GmMT7 (Dhaubhadel et al., 2008).

### GmIMaT1 and GmIMaT3 are Differently Regulated by Various Environmental and Hormonal Factors

**FIGURE 7:**
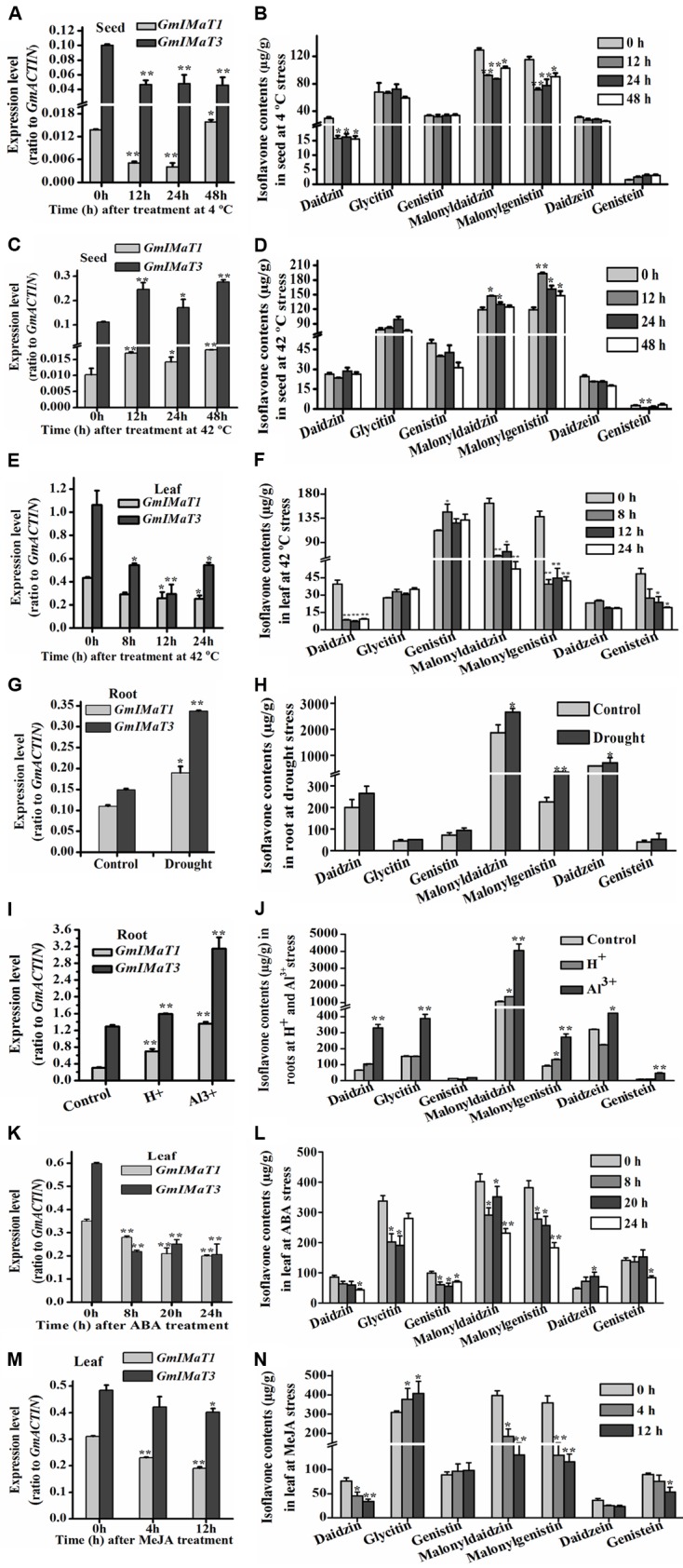
**Altered *GmIMaT* expression and isoflavone profiles in response to stresses**. Differential expression patterns of GmIMaT1 and GmIMaT3 in soybean tissues under various conditions were examined, simultaneously with measurement of isoflavone contents in the same samples. Eight-week-old soybean plants were treated under cold (4°C) **(A,B)** or heat (42°C) **(C–F)** stress and pods **(A–D)** and leaves **(E,F)** were sampled in different time intervals. Six-week-old soybean plants were subjected to drought stress and roots were sampled after 10 days of treatment for analyses **(G,H)**. For acidic condition (pH 4.0) treatment and 50 μM Al^3+^ stresses (under pH 4.0), hydroponically cultivated seedlings were transferred to these media for 10 days before harvesting roots for analysis of both gene expression and isoflavone profiling **(I,J)**. Soybean seedlings with nine trifoliate were sprayed with hormones (100 μM ABA) **(K,L)**, or their detached leaves floated on 50 μM MeJA solution and water (control) **(M,N)**. Data are expressed in means ± SD from at least three independent experiments. Differences between control and treated samples were analyzed by using Student’s *t*-test in two-tailed comparison. ^∗^*P* < 0.05, and ^∗∗^*P* < 0.01.

*GmIMaT1* and *GmIMaT3* also displayed differential expression patterns in response to abiotic stresses, such as heat, drought, and cold stresses, and to hormonal stresses (**Figure 7**). Both *GmIMaT1* and *GmIMaT3* transcripts in developing seeds under cold treatment (4°C) initially decreased at 12 and 24 h. GmIMaT1 transcripts recovered at 48 h, whereas GmIMaT3 did not (**Figure 7A**). Under heat stress at 42°C, GmIMaT1 and GmIMaT3 transcripts in developing seeds showed almost same expression trend: both transcripts increased firstly, then decreased at 24 h, followed by increases at 48 h post-treatment (**Figure 7C**). However, both transcripts in leaves were down-regulated markedly when subjected to heat stress at 42°C (**Figure 7E**). We also analyzed isoflavonoids in these seeds, and leaves under treatments. Upon cold treatment, malonylisoflavone contents showed the same trend as *GmIMaT1* and *GmIMaT3* transcripts. The malonyldaidzin and malonylgenistin contents decreased after 12, 24 h and then slightly increased after 48 h of cold treatment. Daidzein aglycone and daidzein glucoside contents also decreases while other contents remains not changed (**Figure 7B**). Heat stress affected the malonylisoflavone contents in a reverse trend with cold stress. The malonyldaidzin and malonylgenistin contents increase after 12 h, then start to decrease at 24 and 48 h (**Figure 7D**). The trends of isoflavone malonate changes in leaves were consistent with both *GmIMaT* transcript levels: all remarkably decreased after heat treatment of leaves (**Figure 7F**). Both genes were up-regulated in drought-stressed soybean roots than control (**Figure 7G**). The contents of malonyldaidzin and malonylgenistin in roots under drought treatment increase along with daidzein aglycone and daidzein glucoside (**Figure 7H**), which is consistent with GmIMaTs expression in response to drought stress.

A number of transcriptomic and proteomic studies had shown that Al^3+^ stress triggers significant changes in expression of key isoflavone biosynthesis genes, such as *4CL*, *CHS*, and *IFS* in soybean roots (Zhen et al., 2007; You et al., 2011). To investigate whether isoflavone malonyltransferases are also changed under low pH and Al^3+^ stresses, we have treated soybean roots with Al^3+^ stress under low pH conditions to verify their effect on *GmIMaT* expression. Under acidic conditions and Al^3+^ stress in low pH, both GmIMaT1 and GmIMaT3 in roots were up-regulated significantly (*P* < 0.01). In particular, GmIMaT3 was highly up-regulated under Al-stress, as compared with low pH (**Figure 7I**). Isoflavone analyses indicated that Al-stress stimulated the production of the most isoflavones, including daidzein, genistein, and its malonates in roots (**Figure 7J**). Low pH stress only triggered accumulation of daidzin and its malonate, and genistin malonate contents significantly (*P* < 0.05; **Figure 7J**). These data suggest that the low pH and Al stresses evoked isoflavone biosynthesis, most likely as a defense strategy in soybean.

Both *GmIMaT* genes showed same response to hormonal stress. Abscisic acid (ABA) treatment down-regulated both genes after 8, 20, and 24 h leaves treated samples (**Figure 7K**). MeJA also down-regulate both genes like ABA after 4 and 12 h treatment. Both GmIMaTs showed same expression after 0, 4, and 12 h as control samples but decreased expression in MeJA treated leaves (**Figure 7M**). The isoflavone glucoside malonates profiles in ABA-treated leaves were consistent with GmIMaTs expression patterns. Malonyldaidzin and malonylgenistin contents were decreased along with glucosides but aglycones increased after ABA treatment (**Figure 7L**). MeJA also decreased the malonylisoflavone contents in treated leaf as decreased the expression of GmIMaTs. The malonyldaidzin and malonylgenistin contents decreased at 4 and 12 h after MeJA treatment while glycitin contents increased after 4 h of treatment (**Figure 7N**).

## Discussion

The malonylation of isoflavones in soybean is quite common and their isoflavone malonates often take account for majority of total isoflavones in different tissues (Dhaubhadel et al., 2003, 2008). Although one of the major enzymes responsible for the modification, GmMT7/GmIF7MaT, has been characterized in soybean, more details about it and many other isoflavone:malonyl CoA acyltransferase (IMaTs) among more than 100 BAHD family in soybean genome remain unknown (Suzuki et al., 2007; Dhaubhadel et al., 2008; **Supplementary Figure S5**). While *in vitro* activity assay does not always reflect *in planta* functions, here, we not only biochemically characterized the enzyme corresponding to a new allele of GmMT7/GmIF7MaT, IMaT3, and a new BAHD member, IMaT1, and determined their *in vitro* enzyme kinetics, but also investigated their functions in transgenic soybean hairy roots. Furthermore, their putative biological functions were investigated in soybean plants under various conditions, with gene expression profiling, in combination of metabolite analysis of their *in vivo* isoflavone glucoside substrates and malonate products. These studies definitely extend our understanding on the process and role of isoflavone modifications in soybean plants, which provide new insights into soybean isoflavone metabolism and physiological functions.

### GmIMaT1 and GmIMaT3 Catalyze the Malonylation of Isoflavone Glucosides *In Vitro*

Although isoflavone biosynthesis is well studied and genes encoding stepwise structural enzymes have been mostly cloned from leguminous plants, many details about the modification, transport, and storage of flavonoids within the cellular or subcellular compartments remain largely unknown (Zhao, 2015). The majority of the soybean isoflavonoids accumulate in glycosyl and malonyl derivatives, which are presumably through the glycosylation of isoflavonoid aglycones by UGTs and further malonylation of the resulting isoflavone glucosides by IMaTs. The molecular identity of isoflavone malonyltransferases responsible for production of various types of isoflavone glucoside malonates remains elusive. BAHD family of acyltransferases has been characterized for the acyl-CoA-dependent O- or N-acylation of secondary metabolites (Ma et al., 2005; D’Auria, 2006; Suzuki et al., 2007). Two alleles of the same gene GmMT7/GmIF7MaT have been characterized for isoflavonoid glucoside malonate biosynthesis in *G. max* (Suzuki et al., 2007; Dhaubhadel et al., 2008). Both GmIMaT1 and another allele of GmIMT7/GmIF7MaT, GmIMaT3, functioned as isoflavone 7-*O*-glucoside-specific malonyltransferases; they could not use anthocyanins and flavonol glucosides (3-*O*-glucosides) as substrates, suggesting a stereospecific activity. The recombinant GmIMaTs also exclusively uses malonyl CoA, but not acetyl-CoA, as an acyl donor, just like other BAHD members (Suzuki et al., 2007; Dhaubhadel et al., 2008; Yu et al., 2008; Zhao et al., 2011). Medicago MaT5 and MaT6 showed substrate preferences toward only malonyl-CoA as the acyl donor and specificity toward 3-*O*-glucosides as acceptors (Dhaubhadel et al., 2008; Yu et al., 2008). Medicago MaT3 and MaT4 showed the specificity toward 7-*O*-glucosides (Zhao et al., 2011). GmIMaT1 and GmIMaT3 showed similarly wide substrate specificity toward three major soybean isoflavone glucosides daidzin, genistin and glycitin (**Figure 3**). GmIMaT1 and GmIMaT3 showed higher *K*_m_ values than GmIF7MaT1, which were 3.8 and 6.6 μM for daidzin and genistin, respectively (**Table 1**; Suzuki et al., 2007), but lower than GmMT7 that was reported as 68.13 and 39.16 μM, respectively (Dhaubhadel et al., 2008). The *K*_m_ values of GmIMaT1 and GmIMaT3 for genistin were more than four times lower than MtMaT1 (131.4 μM; Yu et al., 2008; **Table 1**). Although GmIMaT1 and GmIMaT3 showed higher *K*_m_ values than GmIF7MaT1 for daidzin and genistin, their *K*_cat_/*K*_m_ values were much lower than these of GmIF7MaT1 (13.68 and 5.45 μM^-1^ S^-1^ for daidzin and genistin, respectively; **Table 1**; Suzuki et al., 2007). These differences may be due to different assay conditions, such as reaction conditions, enzyme purification, and substrates. Both GmIMaTs prefer to use malonyl-CoA as an acyl donor, like other BAHD members (Nakayama et al., 2003; Suzuki et al., 2004, 2007; Dhaubhadel et al., 2008). Although soybean plants also synthesize small portions of isoflavone glucoside acetates, such as acetyldaidzin, acetylgenistin, no acetyltransferase activity were defined for GmIMaT1 and GmIMaT3 in this biochemical analysis. In soybean genome, there are more than 100 BAHD family members (Supplementary Note 3), it is likely that other BAHD gene may have a role for synthesis of these specific types of isoflavone derivatives.

### Subcellular and Tissue Specific Expression of *GmIMaT*s for Isoflavone Modification

The primary subcellular localizations of GmIMaT1 in the ER and GmIMaT3 in the cytosol might reflect their functions after isoflavone glucosides are synthesized, and ready for malonylation. Similar to other flavonoids, the isoflavone glucosides are also believed to be synthesized on the cytosolic side of ER, and then further modified by MaTs (Funaki et al., 2015). The previous studies also showed diverse subcellular localization of MaTs, such as the cytosol (Achnine et al., 2005; Dhaubhadel et al., 2008; Zhao et al., 2011), the ER (Zhao et al., 2011), or the nucleocytoplasm (Yu et al., 2008). The difference in the subcellular localization for GmIMaT1 and GmIMaT3 may account for their different physiological functions in modification of different isoflavones, which then carry on different roles in plant–microbe and plant–insect interactions, or plant adaptation to various environmental stresses.

The functions of GmIMaT1 and GmIMaT3 for production of isoflavone glucoside malonates are also reflected on their tissue specific expression patterns and metabolite profiles. The root and seed are the main stores for malonylisoflavonoids (Graham, 1991); alleles of GmMT7 and GmIMaT3 have higher expression levels in seeds and roots (Suzuki et al., 2007; Dhaubhadel et al., 2008). Roots contain a large amount of isoflavones in various forms; both GmIMaT1 and GmIMaT3 has higher expression levels in root, nodule, and flower, although it is also expressed in all other tissues (**Figure 2B**). It is speculated that the distinct tissue-specific expressions of two GmIMaTs may also imply their different physiological functions, at least for modification of isoflavones for different purposes (Dhaubhadel et al., 2008).

### GmIMaTs Are Responsible for the Malonylation of Soybean Isoflavone Glucosides

The soybean hairy roots overexpression and knockdown of two *GmIMaT1* and *GmIMaT3* also verified the *in planta* activities. The levels of isoflavonoid 7-*O*-glucosides malonates in the transgenic hairy roots were closely associated with the overexpression or knockdown of *GmIMaT1*. The data from overexpression hairy root lines clearly demonstrate the markedly increases in three main isoflavone glucoside malonates as compared with the *GUS* control (**Figure 4**). The increases in aglycones and isoflavonoid glucosides were also observed, probably due to the more rapid conversion of these compounds into the malonate forms may facilitate their transport and storage, thus removing of the feedback inhibition that is a commonly occurred regulatory mechanism in flavonoid biosynthesis (Zhao et al., 2011). On the contrary, the knockdown of *GmIMaT1* in hairy roots did not alter significantly the isoflavone glucoside as compared to *GUS* control; the levels of isoflavone glucosides were almost same or even higher than these in *GUS* control (**Figure 5J**). Since *in vitro* activity assays had shown that glycitin was the favorite substrate for both GmIMaT and GmIMaT3, surprisingly, among varying levels of the malonylated isoflavonoids, i.e., malonyldaidzin, malonylglycitin and malonylgenistin, the malonyldaidzin was found in highest concentration than the others. This inconsistency is probably due to the availability of higher levels of endogenous daidzein as substrate for GmIMaT1 in hairy roots, rather than the *in vitro* enzymatically preferred glycitin for GmIMaTs.

Some other MaT genes were reported to convert the isoflavone 7-*O*-glucosides into their corresponding malonates *in vitro*, such as MtMaT1 and MtMaT4 (Suzuki et al., 2007; Dhaubhadel et al., 2008; Yu et al., 2008; Zhao et al., 2011), there are few relevant studies on *in planta* functions of these MaTs. Ectopic overexpression of *MtMaT1* in Arabidopsis mutant engineered to be able to generate genistein glucoside resulted in the production of malonylgenistin (Yu et al., 2008). However, the *in planta* functions of most other characterized isoflavonoid malonyltransferases were not confirmed, including soybean GmMT7/GmIF7MaT, we here demonstrated the *in planta* functions of GmIMaT1 and GmIMaT3 through their overexpression and RNAi-knockdown chimerical transgenic soybean plants.

### *GmIMaT1* and *GmIMaT3* Differentially Respond to Abiotic, Biotic, and Hormonal Stresses

In soybean genome, at least 100 BAHD acyltransferase family genes can be found, and many of GmIMaT homolog genes are arranged in tandem in chromosomes (Supplementary Note 3). For instance, GmIMaT1 and its homologs arranged in tandem in Chr 18 (**Supplementary Figure S5A**). Most of them are expressed universally at various levels and tissues or under different circumstances (**Supplementary Figure S5B**). The tandem clusters of paralogous genes encoding flavonoid malonyl transferase or BAHD family acyltransferase enzymes suggest their origin from local gene duplication, similar to other isoflavone modification genes (Yoneyama et al., 2016; **Supplementary Figure S5C**). The abundant malonyltransferases in soybean may reflect their necessary functions in modification of various flavonoids or other metabolites and physiological importance for certain processes, although the biological significance of malonylation of flavonoids remains elusive (**Supplementary Figures S5C,D**).

It is often observed that legume roots secret isoflavone aglycones such as genistein and daidzein to function in legume–microbe interactions and other adaptations to changed environments (Dixon, 2001). Soybean tissues infected with pathogens produce isoflavonoid phytoalexins (coumestrol and glyceollins) in site during defense responses to pathogen attacks (Zavala et al., 2015; Nakata et al., 2016; Yoneyama et al., 2016). The fluctuations of endogenous isoflavones in various forms, such as glucosides and malonates, are also detected under various conditions, although their coding physiological meanings barely are understood (Suzuki et al., 2007; Dhaubhadel et al., 2008; Farag et al., 2008; Zhao et al., 2011; Zhao, 2015). As one type of the principal isoflavones in soybean, genistin, daidzin, glycitin and their malonyl conjugates and aglycones, isoflavone glucoside malonates may serve as essential storage forms for regulating the endogenous levels of isoflavones and normal physiological functions in soybean plants (Zhao et al., 2011). As such, GmIMaTs are important and therefore in evolution enriched in soybean genome. Multiple homologs of GmIMaT genes exist in genome and their high expression levels and diverse expression patterns indicate the redundancy of malonylation modification of isoflavonoids in soybean (Supplementary Note 3). The small changes in malonyldaidzin, malonylgenistin, and malonylglycitin contents in *GmIMaT1*- and *GmIMaT3*-knockdown hairy roots further support the redundancy of these GmIMaTs in soybean.

## Conclusion

We show that GmIMaT1 is a new BAHD acyltransferase that shares similar enzymatic kinetics with GmIMaT3/GmMT7/GmIF7MaT alleles in 6′′-*O*-malonylation of several isoflavone 7-*O*-glucosides. The biochemical characters of two recombinant GmIMaTs, such as substrate specificity and enzymatic kinetic parameters, are analyzed. The subcellular localization of GmIMaT1 and GmIMaT3 in the ER or ER- and cytoplasm, respectively, further supported the functions of GmIMaT1 and GmIMaT3 in modification of isoflavone glucosides in soybean. The malonylation activity of GmIMaT1 and GmIMaT3 was confirmed genetically in the *in vitro* transgenic hairy roots. Overexpression or *RNAi*-knockdown GmIMaT1 and GmIMaT3 significantly changed isoflavone profiles, in particular, malonylisoflavonoids, to different extents. However, two malonyltransferase genes display distinct expression patterns in tissue- and organ-specific expression, and different responses to various abiotic and biotic stresses and hormonal factors. The isoflavone metabolite profiles in the corresponding soybean samples mostly coincide with two *GmIMaT* genes’ expression, which may suggest that they play important physiological functions in plant adaptation to environmental stresses and hormonal cues, and that other isoflavone malonyltransferases with redundant functions in modification of isoflavonoids may play complementary roles under stress conditions. These insights provided by this study not only help to understand when these malonylisoflavonoids are synthesized, but also what putative physiological roles of these genes and malonylisoflavonoids in soybean plants in response to various stresses. Further investigations are needed to explore the exact functions of these malonylisoflavonoids in soybean plants under various stress conditions.

## Materials and Methods

### Reagents

All aglycones or conjugated flavones or isoflavone substrates were bought from Dalian Meilun Biological Technology Co. Ltd. (Dalian, Liaoning Province, China). Whereas, malonyl-CoA was purchased from Sigma-Aldrich, St. Louis, MO, United States. The vector pGEM-T easy was purchased from Promega (Madison, WI, United States); the gateway vectors pDONR221, pDEST17, pB2GW7, pB7GWIWGII, and pK7WGF2 were either purchased from Invitrogen (Rockville, MD, United States), or gifts from Dr. Richard Dixon’s Lab at the Noble Foundation, OK, United States.

### Cloning of Malonyltransferase Genes

Soybean roots were used to extract total RNA with TRIzol reagent (Invitrogen, Carlsbad, CA, United States) or from RNA kit (Biotech, Beijing) according to the manufacturer’s instructions. Total RNA (10 μg) were used to synthesize the first-strand cDNA using the Superscript III first strand synthesis system (Invitrogen). The open reading frames (ORFs) of *GmIMaT1* (Glyma.18G268200.1) and *GmIMaT3* (Glyma.13G056100.1) were amplified from the cDNA with the primer pairs listed in Supplementary Table S1. The gel purified PCR products for each gene was inserted in pGEM-T easy vector (Promega, WI, United States) for sequencing. The resultant ORFs for the two genes were subcloned into the entry vector pDONR221 (Invitrogen, Rockville, MD, United States) using BP clonase enzyme, and then were recombined by using LR recombinase into different destination vectors (Invitrogen, Rockville, MD, United States), such as pDEST17 for bacteria expression, pB2GW7 for *in planta* expression, pB7GWIWGII for RNAi constructs, and pK7WGF2 for green fluorescent protein (GFP) fusion constructs.

### Heterologous Expression of Recombinant GmIMaT1, and GmIMaT3

The pDEST17-GmIMaT1 and pDEST17-GmIMaT3 constructs were transformed into *E. coli* strain BL21 (DE3) for heterologous protein induction. The fresh bacteria colony was grown overnight, diluted in 1:100 and grown to OD_600_ to 0.8–1.0 at 37°C in Luria-Bertani (LB) medium containing 50 μg ml^-1^ ampicillin. After adding isopropyl 1-β-D-thiogalactoside (IPTG) at a final concentration of 0.4 mM, the culture was grown at 20°C for another 16 h. The bacteria were harvested by centrifugation and re-suspended in lysis buffer [200 mM Tris–HCl (pH 8.0), 2.5 mM phenylmethane sulfonyl fluoride (PMSF), 5 mM 2-β-mercaptoethanol, 0.1% Triton X-100]. The cells were chilled on ice for 1 h before being broken by ultrasonication. After separation of supernatant from the cell debris with high speed centrifugation at 4°C, the supernatant was purified by using a nickel-resin purification kit. The sodium dodecyl sulfate–polyacrylamide gel electrophoresis (SDS-PAGE) was used to examination of the protein induction and purification.

### Enzyme Assay and HPLC/LC-MS Analyses of Enzymatic Reaction

The malonyltransferase activity of recombinant GmIMaT1 and GmIMaT3 enzymes was assayed in a total reaction mixture (50 μl) consisting of 20 mM potassium phosphate buffer (pH 7.2), 40 μM malonyl-CoA or acetyl-CoA, 80 μM (iso)flavonoid glycoside, and enzyme at 30°C for 30 min. The equal amount of 100% methanol was added to stop the reactions, and the reactions were analyzed on reverse-phase HPLC as described previously (Li et al., 2016). The chromatograms of isoflavone were detected at 254 nm using a SPD M-20A HPLC with a DAD detector (Shimadzu, Kyoto, Japan). Further confirmation of the enzymatic production of malonyl isoflavone glucosides was conducted with tandem LC-MS as described previously (Zhao et al., 2011).

### Enzyme Kinetic Study

The steady-state kinetic parameters (*K*_m_ and *V*_max_) for GmIMaT1 and GmIMaT3 were conducted with various isoflavone glucosides as substrates. The daidzin, genistin, and glycitin concentrations range from 5 to 210 μM with the constant amount of malonyl-CoA (40 μM) and protein were used in a total volume of 50 μl. The kinetics of GmIMaT1 and GmIMaT3 for malonyl-CoA were also measured by using various concentrations (5–180 μM) of acyl donors and a fixed concentration (60 μM) of genistin as an acyl acceptor in a total reaction volume of 50 μl. All the reactions were incubated at 30°C for 20 min. The other reaction conditions were same as described above. The product was assayed on HPLC. The *K*_m_ and *V*_max_ values were calculated from Lineweaver–Burk plot.

### Soybean Plant Treatment for Gene Expression and Isoflavone Metabolite Analyses

Soybean seedlings were treated under cold and heat, or sprayed with ABA (at 100 μM) or their leaves floated on MeJA (at 50 μM) solution or water (as a control) as described previously (Chen et al., 2016). Briefly, 8-week-old soybean plants were treated under cold (4°C) or heat (42°C) stress in incubators. Leaves or (seeds) were harvested at different time intervals. Soybean seedlings with nine trifoliate were subjected to spraying with hormones (100 μM ABA), or their detached leaves were floated on 50 μM MeJA solution and water (control). Six-week-old soybean plants were subjected to drought stress and roots were harvested after 10 days of treatment for analyses. For acidic condition (pH 4.0) treatment and 50 μM Al^3+^ stress (under pH 4.0; Wang et al., 2017), hydroponically cultivated seedlings were transferred to these media for 10 days before harvesting roots for analysis of gene expression and isoflavones (Li et al., 2016).

### Gene Expression Analysis Using Quantitative Reverse Transcriptase PCR

Total RNA was isolated from different *G. max* organs or tissues (seeds, root, nodules, stem, leaf, flower, and pod) with TRIzol reagent or RNA isolation kit and quantified by using NanoDrop Spectrophotometer (Thermo Fisher Scientific, Yokohama, Japan). The first strand cDNA was synthesized by using the Superscript III first strand synthesis system (Invitrogen). qRT-PCR was performed with gene specific primers (Supplementary Table S1). The total reaction mixture (20 μl) consists of 2.5 μl of a Power SYBR Master Mix (Applied Biosystems), 1 μl of primer mix (0.4 μl of each primer, 0.2 μl H_2_O) and 2 μl of 100 ng cDNA in 96-well plates with iQ5 Real Time PCR System (Bio-Rad). Housekeeping genes *GmACTIN* (Glyma19G147900.1) was used to normalize the transcript levels.

### Overexpression and RNAi Knockdown of *GmIMaT1* and *GmIMaT3* in Soybean Hairy Roots

GmIMaT1 and GmIMaT3 were constructed in pB2WG7 for overexpression and pB7GWIWGII for RNAi and GUS as control. These confirmed constructs were transformed into *Agrobacterium rhizogenes* strains K599 by electroporation. The positive colonies were selected on LB-agar medium containing selective antibiotics at 28°C. Positive K599 colonies were used to generate the hairy roots from *G. max* cotyledons (Chen et al., 2016). Soybean cultivar “Tianlong #1” seeds were surface sterilized and germinated in the Petri dishes containing sterilized filter paper. The surface of about 7 days old green cotyledons were wounded and infected with K599 harboring constructed vector for overexpression and RNAi. The overexpression and RNAi-knockdown transgenic hairy roots were subjected to semi- or qRT-PCR analyses for verification of genetic backgrounds. The transgenic hairy roots were maintained on a half-strength Murashige and Skoog medium (MS medium) containing 2.5 mg l^-1^ phosphinothricin (ppt) for selection in a growth chamber at 23°C with 16 h/8 h of light/dark photoperiod and subcultured every 3–4 weeks. HPLC analyses for isoflavone profile.

### Analysis of Isoflavones in Transgenic Soybean Hairy Roots

HPLC analyses of overexpressed and knockdown hairy roots were done to evaluate the function of GmIMaT1 and GmIMaT3 as malonyltransferase gene. The transformed hairy roots were collected from selection medium and grinded into powder in liquid nitrogen. About 100 mg fresh weights of hairy roots powder were taken and dissolved in 1 ml 80% methyl alcohol followed by sonication for 30 min. Samples were kept on rotator shaker for overnight at 4°C. The transparent liquid layer was taken after centrifugation at 12,000 rpm for 30 min at 4°C and analyzed for isoflavonoids detection by using reverse-phase HPLC. The authentic standard samples of each isoflavone were also used to confirm the compound in the sample. Concentration of isoflavone was calculated using the standard curve.

### Subcellular Localization of GmIMaT1 and GmIMaT3

Both GmIMaT1 and GmIMaT3 were recombined into pK7WGF2 in fusion with GFP at N-terminal by using Gateway recombination technology. Resulting GFP-GmIMaT1 and GFP-GmIMaT3 were transformed into *Agrobacterium tumefaciens* strain EHA105 through electroporation. Tobacco leaf infiltration was performed with these transformed Agrobacteria to determine the subcellular localization of GFP-GmIMaT1 and GFP-GmIMaT3 as described previously (Zhao et al., 2011). The acetosyringone were used as an activator of Agrobacteria infiltrated with a syringe into the abaxial epidermal surface of a tobacco leaf. The GFP-GmIMaT1, GFP-GmIMaT3, and ER marker CD3-959:mCherry-infiltrated plants were grown at room temperature for 3–4 days. A Leica TCS SP2 confocal microscope was used for imaging of fusion proteins. Leica confocal software with an excitation wavelength of 488 nm and emissions collected at 500 nm. ER membrane labeled CD3-959:mCherry marker was animated at 543 nm with argon laser and emission was detected from 620 to 680 nm.

### Bioinformatics Analysis

GmIMaT1 and GmIMaT3 were aligned with other characterized malonyltransferases from other plants by using multiple protein sequence alignment software ClustalW^1^. A phylogenetic tree was constructed by using MEGA6. The significance level of the neighbor-joining analysis was examined by bootstrap testing with 1000 repeats. The sequence similarities and identities were determined from Blast2^2^.

Proteins and GenBank accession numbers are as follow: *G. max* GmIMaT1 (KY399789); GmIMaT3 (KY399790); GmMT7 (EU192928.1); GmIF7MaT (BAF73620); GmACTIN (XM_014771363.1,Glyma.19G147900.1); *M. truncatula* MtMaT1, MtMaT2, MtMaT3, MtMaT4, MtMaT5, and MtMaT6 (EU272030.1, EU272032.1, EU272031.1, HM856606.1, HM856607.1, ADV04048.1, respectively), *Arabidopsis thaliana* At5MaT (OAP06471.1), *Verbena* ×*hybrida* Vh3MAT1 (AY500350.1), *Lamium purpureum* Lp3MAT1 (AAS77404), *Nicotiana tabacum* NtMAT1 (BAD93691), *Salvia splendens* Ss5MaT1 (AAL50565), Ss5MaT2 (Q6TXD2.1), *Dahlia pinnata* Dp3MAT (Q8GSN8), *Chrysanthemum × morifolium* Cm3MaT1 (AY298809), Cm3MaT2 (AY298810), and Cm3MaT3 (BAF50706).

### Statistic Analysis

In most cases, at least three independent experiments with duplicates were performed. Differences between paired data from the mutants (overexpression or knockdown hairy roots) and the GUS control under various conditions were analyzed by using Student’s *t*-test (*n* = 3). ^∗^*P* < 0.05, ^∗∗^*P* < 0.01. The differences between two-tailed data with the error bars represent 95% confidence limits.

## Author Contributions

JZ planned and designed the research. MA, PL, JW, and NR performed experiments. MA and PL conducted metabolite analyses. JZ and MA wrote the manuscript.

## Conflict of Interest Statement

The authors declare that the research was conducted in the absence of any commercial or financial relationships that could be construed as a potential conflict of interest.

## Footnotes

1 http://www.genome.jp/tools/clustalw/

2 http://danio.mgh.harvard.edu/blast/wblast2.html

## Supplementary Material

The Supplementary Material for this article can be found online Q2 at: http://journal.frontiersin.org/article/10.3389/fpls.2017.00735/full#supplementary-material

FIGURE S1 **(a)** Sequence alignment of GmIMaT1 and GmIMaT3 with MtMaT1. Amino acid sequence alignment of GmIMaT1 and GmIMaT3 with MtMaT1 was done with MEGA6 GeneDoc program. Three conserved motifs among these BAHD family protein sequences are highlighted with red underlines. Dark shade represents identical amino acids and gray shade indicates similar amino acids. **(b)** Sequence alignment of GmIMaT3 with GmMT7 and GmIF7MaT. MEGA6 program was used for alignment and the GeneDoc program used to shade the identical and similar amino acids in alignment. Dark shade represents identical amino acids and gray shade indicates similar amino acids.

FIGURE S2 ***GmIMaT1* and *GmIMaT3* expression patterns in soybean tissues. (a,b)**. Expression patterns of *GmIMaT1* in different tissues of soybean plant. Data are retrieved from public database phytozome (https://phytozome.jgi.doe.gov/) for GmIMaT1 (Glyma.18G268200.1 in v9 or Glyma18g50320 in v10). **(c)**. *GmIMaT1* is mainly expressed in nodule, flower, root, and lower expression in seed, but maximum in nodule. These data are retrieved from soyKB (http://soykb.org/). **(d)**. Expression patterns of *GmIMaT1* in root tissues infected with rhizobial bacteria for different times, as compared with other tissues. *GmIMaT1* was initially up-regulated at 12 h post inoculation, and then repressed upon rhizobial bacteria infection (IN) as compared with uninfection (UN). Data are retrieved from soyKB. **(e,f)**. Expression patterns of *GmIMaT3* in different tissues of soybean plant. The data are retrieved from publica database in phytozome (https://phytozome.jgi.doe.gov/) for GmIMaT3 (Glyma.13G056100.1 in v9 or Glyma13g06230 in v10). **(g)**. *GmIMaT3* is mainly expressed in nodule, root, seed, and lower in flower. Expression level of *GmIMaT3* increases steadily during the seed development. These data are retrieved from soyKB (www.SoyKB.org). **(h)**. Expression patterns of *GmIMaT3* in root tissues infected with rhizobial bacteria for different times. *GmIMaT3* is repressed upon rhizobial bacteria infection (IN) as compared with uninfection (UN). Data are retrieved from soyKB.

FIGURE S3 **The optimization of pH for GmIMaT1 in malonylation of genistin. HPLC analysis of recombinant GmIMaT1-catalyzed malonylation reactions under various pH values**. Genistin was used as an acceptor and malonyl-CoA as an acyl-CoA donor in the reaction. Other conditions were the same as described in “Materials and Methods” Section. The pH range of 6.7–7.5 were adjusted by using appropriate buffers. Data were expressed in average areas (malonylgenistin peaks) ± SD from three independent reactions. Statistix8.1 was used for statistical analyses. One-way ANOVA was used for significance among different pH levels. Lettering was made on LSD basis at *P* < 0.05.

FIGURE S4 **Kinetics of GmIMaT1- or 3-catalyzed isoflavone glucoside malonylation**. Lineweaver–Burk plot were generated to calculate the *K*m and *V*max for GmIMaT1 and 3 in malonylation reactions by using isoflavone glucosides at different concentrations (5 μM to 200 μM). **(a)** Kinetics for malonylation of daidzein 7-*O* glucoside by GmIMaT1. **(b)** Kinetics for malonylation of genistein 7-*O* glucoside with GmIMaT1. **(c)** Kinetics for malonylation of glycitein 7-*O* glucoside with GmIMaT1. **(d)** Kinetics for malonylation of daidzin 7-*O* glucoside with GmIMaT3. **(e)** Kinetics for malonylation of genistein 7-*O* glucoside with GmIMaT3. **(f)** Kinetics for malonylation of glycitein 7-*O* glucoside with GmIMaT3.

FIGURE S5 **Genomic analysis of GmIMaT1 homologs in soybean genome. (A)** GmIMaT1 (Glyma.18G268200) and its homologs are arranged tandem in the Chromosome 18. **(B)** Expression patterns of *GmIMaT1* (Glyma.18G268200) and its homologs in soybean tissues. **(C)** Phylogenetic tree for these GmIMaT1 (Glyma.18G268200) homologs and GmIMaT3 (Glyma.13G056100) with its alleles. **(D)** Phylogenetic analysis of BAHD gene family in soybean genome. Red dot indicate the GmIMaT1 (Glyma.18G268200) and GmIMaT3 (Glyma.13G056100).
