# Supplementary figures and images for: Isoflavone Malonyltransferases GmIMaT1 and GmIMaT3 Differently Modify Isoflavone Glucosides in Soybean (Glycine max) under Various Stresses
Source: Front Plant Sci. 2017 May 16;8:735. doi: 10.3389/fpls.2017.00735 (PMC5433297; doi:10.3389/fpls.2017.00735)

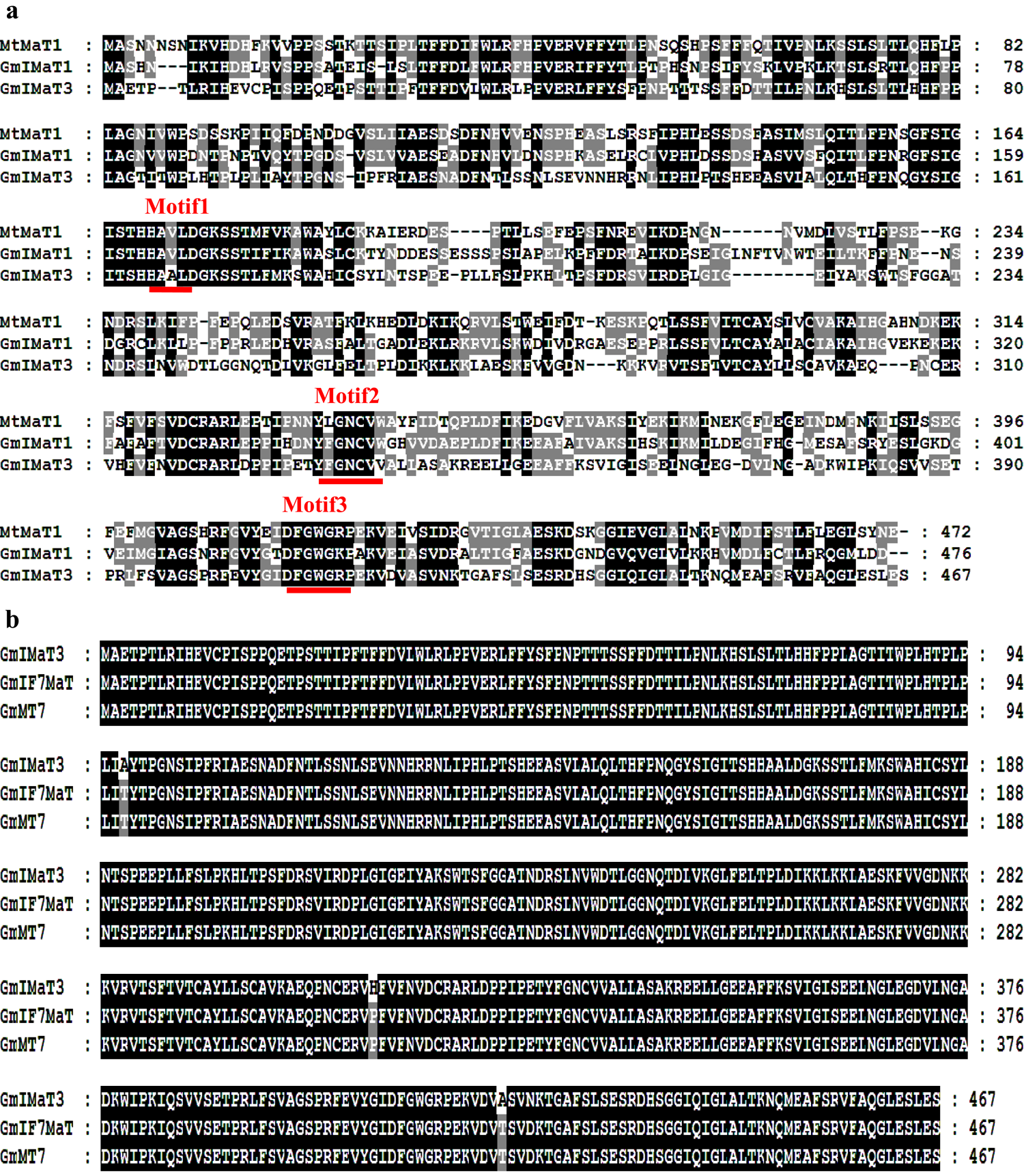

Supplement: FIGURE S1 — (a) Sequence alignment of GmIMaT1 and GmIMaT3 with MtMaT1. Amino acid sequence alignment of GmIMaT1 and GmIMaT3 with MtMaT1 was done with MEGA6 GeneDoc program. Three conserved motifs among these BAHD family protein sequences are highlighted with red underlines. Dark shade represents identical amino acids and gray shade indicates similar amino acids. (b) Sequence alignment of GmIMaT3 with GmMT7 and GmIF7MaT. MEGA6 program was used for alignment and the GeneDoc program used to shade the identical and similar amino acids in alignment. Dark shade represents identical amino acids and gray shade indicates similar amino acids. [file Image_1.TIF]

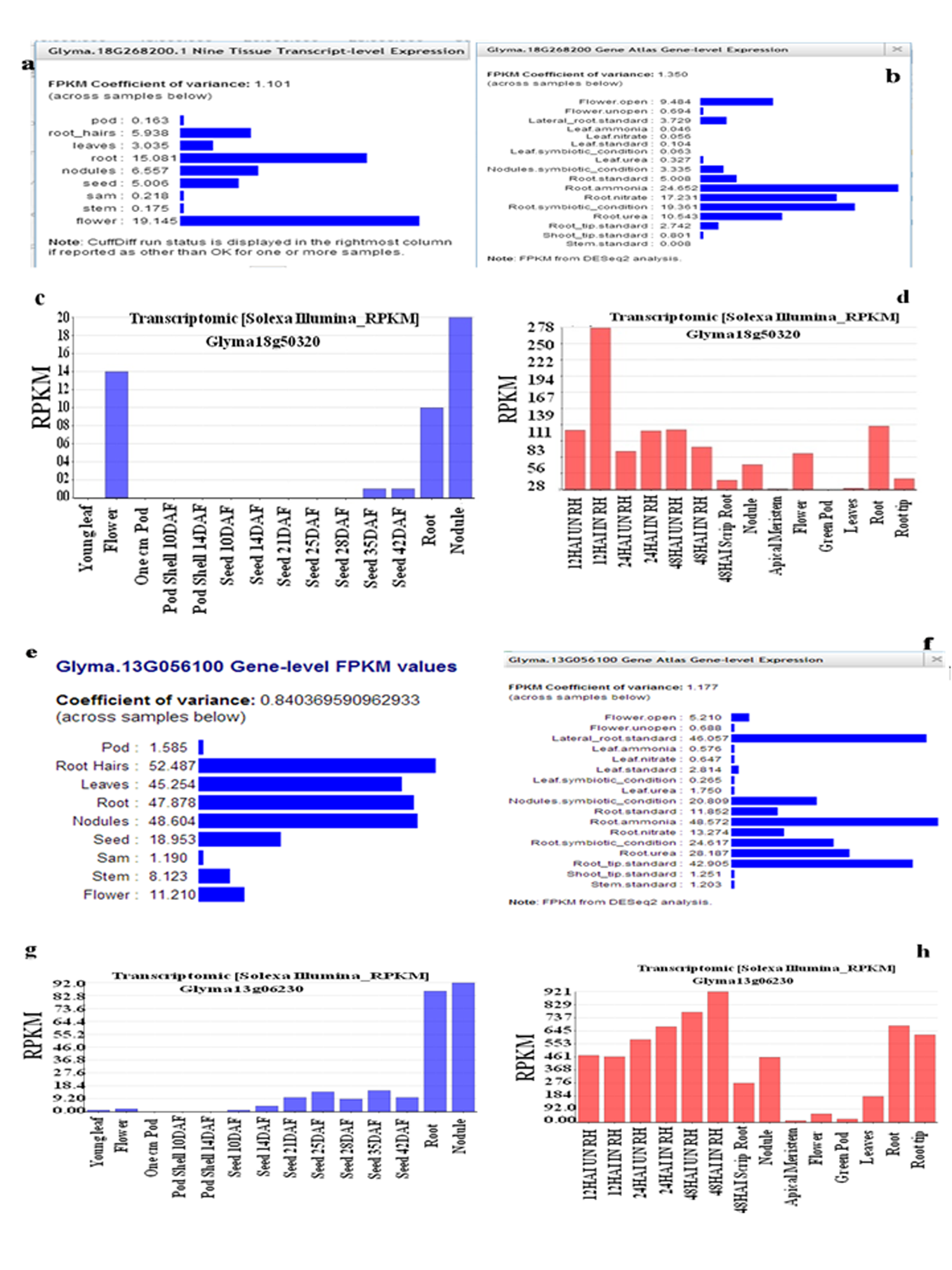

Supplement: FIGURE S2 — GmIMaT1 and GmIMaT3 expression patterns in soybean tissues. (a,b). Expression patterns of GmIMaT1 in different tissues of soybean plant. Data are retrieved from public database phytozome (https://phytozome.jgi.doe.gov/) for GmIMaT1 (Glyma.18G268200.1 in v9 or Glyma18g50320 in v10). (c). GmIMaT1 is mainly expressed in nodule, flower, root, and lower expression in seed, but maximum in nodule. These data are retrieved from soyKB (http://soykb.org/). (d). Expression patterns of GmIMaT1 in root tissues infected with rhizobial bacteria for different times, as compared with other tissues. GmIMaT1 was initially up-regulated at 12 h post inoculation, and then repressed upon rhizobial bacteria infection (IN) as compared with uninfection (UN). Data are retrieved from soyKB. (e,f). Expression patterns of GmIMaT3 in different tissues of soybean plant. The data are retrieved from publica database in phytozome (https://phytozome.jgi.doe.gov/) for GmIMaT3 (Glyma.13G056100.1 in v9 or Glyma13g06230 in v10). (g). GmIMaT3 is mainly expressed in nodule, root, seed, and lower in flower. Expression level of GmIMaT3 increases steadily during the seed development. These data are retrieved from soyKB (www.SoyKB.org). (h). Expression patterns of GmIMaT3 in root tissues infected with rhizobial bacteria for different times. GmIMaT3 is repressed upon rhizobial bacteria infection (IN) as compared with uninfection (UN). Data are retrieved from soyKB. [file Image_2.TIF]

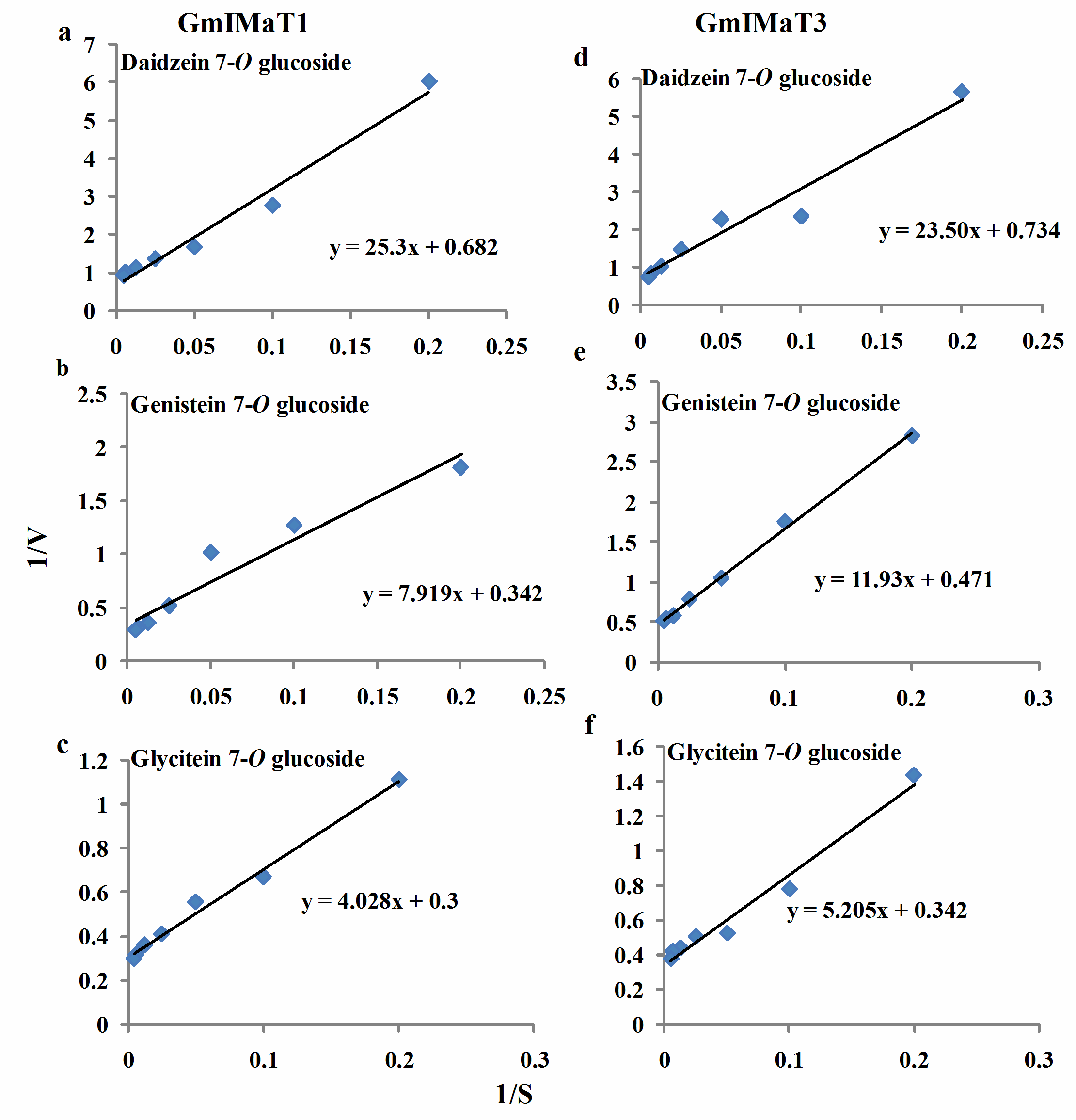

Supplement: FIGURE S4 — Kinetics of GmIMaT1- or 3-catalyzed isoflavone glucoside malonylation. Lineweaver–Burk plot were generated to calculate the Km and Vmax for GmIMaT1 and 3 in malonylation reactions by using isoflavone glucosides at different concentrations (5 μM to 200 μM). (a) Kinetics for malonylation of daidzein 7-O glucoside by GmIMaT1. (b) Kinetics for malonylation of genistein 7-O glucoside with GmIMaT1. (c) Kinetics for malonylation of glycitein 7-O glucoside with GmIMaT1. (d) Kinetics for malonylation of daidzin 7-O glucoside with GmIMaT3. (e) Kinetics for malonylation of genistein 7-O glucoside with GmIMaT3. (f) Kinetics for malonylation of glycitein 7-O glucoside with GmIMaT3. [file Image_4.TIF]

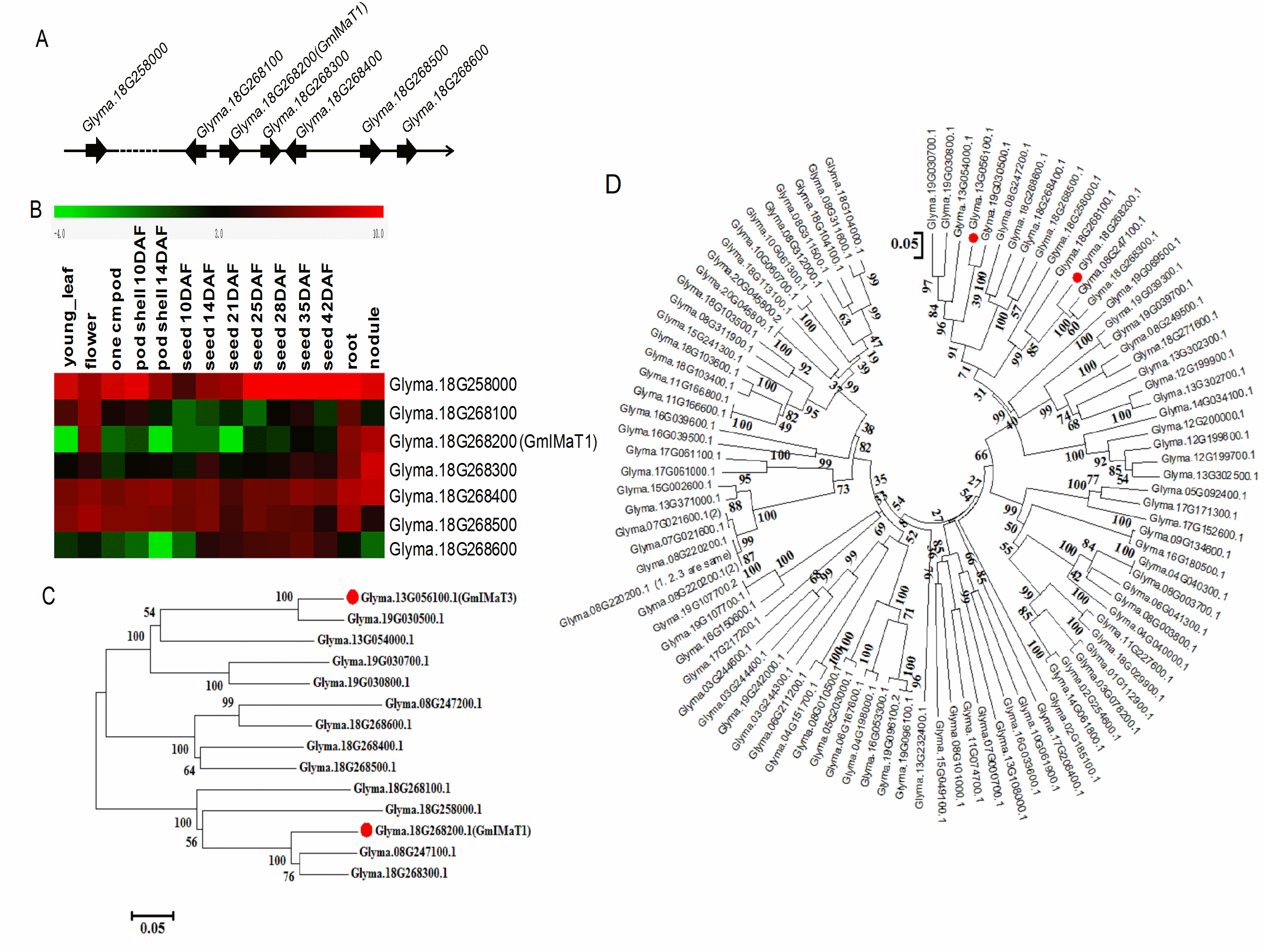

Supplement: FIGURE S5 — Genomic analysis of GmIMaT1 homologs in soybean genome. (A) GmIMaT1 (Glyma.18G268200) and its homologs are arranged tandem in the Chromosome 18. (B) Expression patterns of GmIMaT1 (Glyma.18G268200) and its homologs in soybean tissues. (C) Phylogenetic tree for these GmIMaT1 (Glyma.18G268200) homologs and GmIMaT3 (Glyma.13G056100) with its alleles. (D) Phylogenetic analysis of BAHD gene family in soybean genome. Red dot indicate the GmIMaT1 (Glyma.18G268200) and GmIMaT3 (Glyma.13G056100). [file Image_5.TIF]
