# Supplementary material for: Isoflavone Malonyltransferases GmIMaT1 and GmIMaT3 Differently Modify Isoflavone Glucosides in Soybean (Glycine max) under Various Stresses
Source: Front Plant Sci. 2017 May 16;8:735. doi: 10.3389/fpls.2017.00735 (PMC5433297; doi:10.3389/fpls.2017.00735)
Supplement: Supplementary file 6 [file Data_Sheet_1.DOC]

Supplementary Table S1. List of primers used in this study

| **Primer Name** | **Primer Sequence** | **Purpose** |
| --- | --- | --- |
| GmIMaT1-F | GATGGCTTCTCACAACATCAAAAT | For cloning cDNA into T-easy vector |
| GmIMaT1-R | GCGTCAATCATCTAACATTCCTTG |
| GmIMaT3-F | ATGGCAGAGACACCAACCCT |
| GmIMaT3-R | TCATGATTCCAAGGACTCAAGTC |
| GmIMaT1-F | GGGGACAAGTTTgtacaaaaaagcaggctTCATGGCTTCTCACAACATCAAAAT | For cloning into pDONOR |
| GmIMaT1-R | GGGGACCACTTTGTACAAGaaagctgggtATCAATCATCTAACATTCCTTG |
| GmIMaT3-F | GGGGACAAGTTTgtacaaaaaagcaggctTCATGGCAGAGACACCAACCCT |
| GmIMaT3-R | GGGGACCACTTTGTACAAGaaagctgggtATCATGATTCCAAGGACTCAAGTC |
| GmIMaT1-F | GGGGACAAGTTTgtacaaaaaagcaggctTCGACTCCGTTTCACTTGTGGTTG | For cloning into RNAi vector |
| GmIMaT1-R | GGGGACCACTTTGTACAAGaaagctgggtAAGTCTTGGAGGGAAAGGTAGGA |
| GmIMaT3-F | GGGGACAAGTTTgtacaaaaaagcaggctTCTCACTACCGAAGCATCTAACACC |
| GmIMaT3-R | GGGGACCACTTTGTACAAGaaagctgggtATCAACTCCTCGCTTATCCCTAT |
| GmIMaT1-F | ATAGAGGAGCAGAATCAGAGCC | qRT-PCR |
| GmIMaT1-R | CAATCCACTGTGAACGCAAA |
| GmIMaT3-F | TAACAACCACCGCCGAAAC |
| GmIMaT3-R | ACAAATATGCGCCCACGAT |
| GmACTIN-F | CTTCCCTCAGCACCTTCCAA |
| GmACTIN-R | GGTCCAGCTTTCACACTCCAT |

**Supplementary Table S2. Protein sequence identity and similarity among different malonyltransferase genes**

|  | **GmIMT-1** | **GmIMT-3** | **GmIF7MaT** | **GmMT7** | **MtMaT1** | **MtMaT2** | **MtMaT3** | **MtMaT4** | **MtMaT5** | **MtMaT6** | **At5MaT** |
| --- | --- | --- | --- | --- | --- | --- | --- | --- | --- | --- | --- |
| **GmIMT-1** | **100%** | **58%** | **58%** | **58%** | **73%** | **76%** | **69%** | **73%** | **48%** | **48%** | **56%** |
| **GmIMT-3** | **40%** | **100%** | **99%** | **99%** | **59%** | **59%** | **55%** | **58%** | **52%** | **51%** | **56%** |
| **GmIF7MaT** | **40%** | **99%** | **100%** | **100%** | **59%** | **59%** | **56%** | **59%** | **51%** | **51%** | **56%** |
| **GmMT7** | **40%** | **99%** | **100%** | **100%** | **59%** | **59%** | **56%** | **59%** | **51%** | **51%** | **56%** |
| **MtMaT1** | **57%** | **43%** | **43%** | **43%** | **100%** | **84%** | **74%** | **83%** | **53%** | **51%** | **55%** |
| **MtMaT2** | **62%** | **42%** | **42%** | **42%** | **73%** | **100%** | **71%** | **82%** | **51%** | **49%** | **53%** |
| **MtMaT3** | **53%** | **35%** | **36%** | **36%** | **59%** | **56%** | **100%** | **67%** | **47%** | **46%** | **50%** |
| **MtMaT4** | **58%** | **41%** | **42%** | **42%** | **73%** | **73%** | **55%** | **100%** | **49%** | **50%** | **51%** |
| **MtMaT5** | **32%** | **35%** | **35%** | **35%** | **37%** | **35%** | **32%** | **32%** | **100%** | **52%** | **47%** |
| **MtMaT6** | **31%** | **34%** | **34%** | **34%** | **33%** | **31%** | **28%** | **33%** | **32%** | **100%** | **45%** |
| **At5MaT** | **37%** | **36%** | **36%** | **36%** | **37%** | **36%** | **33%** | **34%** | **31%** | **29%** | **100%** |

Red colour values showing identity and green colour showing similarity among protein sequences

The sequence identity and similarity were calculated through <http://danio.mgh.harvard.edu/blast/wblast2.cgi?1>

**Supplemental Note_1.** cDNA and Protein sequences of GmIMaT1 and GmIMaT3 after sequencing.

**Glyma.18G268200.1 (GmIMaT1)**

ATGGCTTCTCACAACATCAAAATCCACGACCACTTAAGGGTTTCCCCTCCCTCAGCAACAGAAATATCCCTCTCTCTCACTTTCTTCGACCTGTTCTGGCTCAGGTTCCACCCCGTGGAACGCATCTTCTTCTACACCCTCCCTACACCCCATTCAAATCCATCCATTTTCTATTCCAAACTTGTTCCAAAGCTCAAAACATCTCTCTCTCGCACTCTCCAACACTTCCCCCCTCTCGCCGGCAACGTCGTTTGGCCTGATAACACCCCAAACCCCACCGTCCAATACACCCCAGGGGACTCCGTTTCACTTGTGGTTGCTGAATCCGAAGCTGATTTCAACCACGTGTTAGATAACTCACCTCACAAAGCATCAGAGTTACGTTGTTTAGTACCCCACTTGGATTCATCGGATTCTCATGCTTCTGTTGTCTCTTTCCAAATCACTCTGTTCCCTAACAGAGGCTTCAGCATAGGAATCAGCACCCACCATGCCGTCCTTGATGGAAAATCTTCAACTATTTTCATCAAGGCTTGGGCTTCTCTATGCAAAACGTACAATGATGATGAGTCTTCAGAGTCATCATCACCATCTTTGGCTCCAGAGTTGAAGCCTTTCTTTGATAGAACAGCCATCAAAGACCCAAGTGAGATAGGACTTAACTTCACTGTCAATTGGACTGAGATCTTAACCAAATTTTTCCCCAATGAAAACAGCGACGGGAGATGCTTGAAGCTCCTACCTTTCCCTCCAAGACTCGAGGATCATGTTCGAGCCTCGTTCGCGCTCACAGGAGCAGATTTGGAGAAGCTAAGGAAAAGGGTGTTGTCCAAATGGGACATCGTTGATAGAGGAGCAGAATCAGAGCCACCTAGGTTGTCATCTTTCGTTCTCACATGTGCTTATGCGCTAGCTTGCATTGCTAAGGCCATTCATGGAGTTGAAAAGGAGAAAGAGAAATTTGCTTTTGCGTTCACAGTGGATTGCAGGGCGAGGTTGGAGCCTCCAATCCATGATAATTATTTTGGCAATTGTGTGTGGGGGCATGTGGTGGATGCTGAACCATTGGACTTCATAAAGGAAGAAGCTTTTGCTATTGTTGCAAAGAGTATTCATAGTAAAATAAAGATGATATTAGATGAGGGGATTTTTCATGGGATGGAGAGTGCGTTTTCTAGATATGAGTCTTTGGGAAAAGACGGAGTTGAAATCATGGGAATTGCAGGGTCTAACCGGTTTGGAGTTTATGGAACTGATTTTGGTTGGGGAAAGCCTGCTAAGGTGGAGATAGCATCGGTGGATAGAGCCTTAACCATTGGGTTTGCAGAGAGCAAGGATGGGAATGATGGTGTTCAAGTTGGGCTCGTGCTGAAGAAACATGTCATGGATCTCTTTTGTACTTTGTTTCGTCAAGGAATGTTAGATGATTGA

**Glyma.13G056100.1 (GmIMaT3)**

ATGGCAGAGACACCAACCCTCAGAATCCACGAAGTCTGCCCCATCTCGCCGCCACAAGAAACCCCATCAACCACTATTCCCTTCACCTTCTTCGACGTCCTATGGCTACGCCTCCCCCCAGTGGAGCGTCTGTTCTTCTATTCCTTCCCAAACCCAACAACAACCTCTTCATTCTTCGACACCACCATTCTCCCAAATCTCAAACACTCCCTCTCCCTCACTCTCCACCACTTCCCTCCTCTCGCCGGCACCATCACATGGCCACTTCACACACCCCTCCCCCTCATCGCCTACACCCCCGGAAACTCAATCCCCTTCAGAATCGCCGAATCCAACGCAGACTTCAACACCCTCTCTTCAAACCTCTCAGAAGTTAACAACCACCGCCGAAACCTAATACCCCACTTACCCACTTCCCACGAAGAAGCTTCGGTGTTAGCCCTTCAACTCACCCACTTCCCAAACCAAGGCTATTCGATAGGAATAACAAGCCACCACGCAGCACTTGATGGAAAGTCTTCAACTTTGTTCATGAAATCGTGGGCGCATATTTGTTCTTACCTCAATACCTCACCGGAAGAACCGTTGCTGTTTTCACTACCGAAGCATCTAACACCTTCGTTTGATAGATCTGTCATAAGAGACCCTTTGGGGATCGGTGAGATTTACGCGAAGTCGTGGACGAGCTTCGGTGGAGCCACCAATGACCGAAGCTTGAACGTGTGGGATACCCTCGGTGGAAATCAAACCGATTTGGTTAAAGGATTGTTTGAGTTGACACCGTTGGATATCAAGAAGCTGAAGAAGTTAGCGGAGTCCAAGTTTGTTGTCGGAGACAACAAGAAGAAAGTTAGGGTGACATCATTTACGGTCACGTGCGCTTACCTGTTGTCATGCGCGGTGAAAGCGGAGCAACCCAACTGCGAAAGAGTGCATTTTGTCTTCAACGTGGACTGTAGGGCGCGTTTGGACCCCCCAATTCCGGAAACGTACTTCGGGAACTGCGTCGTGGCTTTGTTGGCTTCGGCCAAGCGAGAAGAGCTTTTGGGGGAAGAAGCGTTTTTCAAAAGCGTTATAGGGATAAGCGAGGAGTTGAACGGGTTAGAGGGTGACGTGTTGAACGGCGCGGACAAATGGATTCCGAAGATTCAATCGGTGGTATCGGAGACTCCTAGGTTGTTCTCCGTCGCCGGGTCCCCGAGGTTTGAGGTTTACGGCATTGACTTTGGGTGGGGAAGGCCTGAGAAAGTGGATGTCGCATCCGTTAATAAAACGGGTGCGTTTTCGCTCTCGGAGAGTAGGGATCATAGTGGAGGGATTCAAATTGGGTTGGCGTTGACCAAGAATCAAATGGAGGCGTTTTCTAGGGTTTTTGCTCAAGGACTTGAGTCCTTGGAATCATGA

**Gene Sequencing result (Protein)**

**Glyma.18G268200.1 (GmIMaT1)**

MASHNIKIHDHLRVSPPSATEISLSLTFFDLFWLRFHPVERIFFYTLPTPHSNPSIFYSKLVPKLKTSLSRTLQHFPPLAGNVVWPDNTPNPTVQYTPGDSVSLVVAESEADFNHVLDNSPHKASELRCLVPHLDSSDSHASVVSFQITLFPNRGFSIGISTHHAVLDGKSSTIFIKAWASLCKTYNDDESSESSSPSLAPELKPFFDRTAIKDPSEIGLNFTVNWTEILTKFFPNENSDGRCLKLLPFPPRLEDHVRASFALTGADLEKLRKRVLSKWDIVDRGAESEPPRLSSFVLTCAYALACIAKAIHGVEKEKEKFAFAFTVDCRARLEPPIHDNYFGNCVWGHVVDAEPLDFIKEEAFAIVAKSIHSKIKMILDEGIFHGMESAFSRYESLGKDGVEIMGIAGSNRFGVYGTDFGWGKPAKVEIASVDRALTIGFAESKDGNDGVQVGLVLKKHVMDLFCTLFRQGMLDD

**Glyma.13G056100.1 (GmIMaT3)**

MAETPTLRIHEVCPISPPQETPSTTIPFTFFDVLWLRLPPVERLFFYSFPNPTTTSSFFDTTILPNLKHSLSLTLHHFPPLAGTITWPLHTPLPLIAYTPGNSIPFRIAESNADFNTLSSNLSEVNNHRRNLIPHLPTSHEEASVLALQLTHFPNQGYSIGITSHHAALDGKSSTLFMKSWAHICSYLNTSPEEPLLFSLPKHLTPSFDRSVIRDPLGIGEIYAKSWTSFGGATNDRSLNVWDTLGGNQTDLVKGLFELTPLDIKKLKKLAESKFVVGDNKKKVRVTSFTVTCAYLLSCAVKAEQPNCERVHFVFNVDCRARLDPPIPETYFGNCVVALLASAKREELLGEEAFFKSVIGISEELNGLEGDVLNGADKWIPKIQSVVSETPRLFSVAGSPRFEVYGIDFGWGRPEKVDVASVNKTGAFSLSESRDHSGGIQIGLALTKNQMEAFSRVFAQGLESLES

**Supplemental Note 2.** Protein sequences used for the phylogenetic analysis in Figure 1A. (in FASTA format):

>GmIMaT1

MASHNIKIHDHLRVSPPSATEISLSLTFFDLFWLRFHPVERIFFYTLPTPHSNPSIFYSKLVPKLKTSLSRTLQHFPPLAGNVVWPDNTPNPTVQYTPGDSVSLVVAESEADFNHVLDNSPHKASELRCLVPHLDSSDSHASVVSFQITLFPNRGFSIGISTHHAVLDGKSSTIFIKAWASLCKTYNDDESSESSSPSLAPELKPFFDRTAIKDPSEIGLNFTVNWTEILTKFFPNENSDGRCLKLLPFPPRLEDHVRASFALTGADLEKLRKRVLSKWDIVDRGAESEPPRLSSFVLTCAYALACIAKAIHGVEKEKEKFAFAFTVDCRARLEPPIHDNYFGNCVWGHVVDAEPLDFIKEEAFAIVAKSIHSKIKMILDEGIFHGMESAFSRYESLGKDGVEIMGIAGSNRFGVYGTDFGWGKPAKVEIASVDRALTIGFAESKDGNDGVQVGLVLKKHVMDLFCTLFRQGMLDD

>GmIMaT3

MAETPTLRIHEVCPISPPQETPSTTIPFTFFDVLWLRLPPVERLFFYSFPNPTTTSSFFDTTILPNLKHSLSLTLHHFPPLAGTITWPLHTPLPLIAYTPGNSIPFRIAESNADFNTLSSNLSEVNNHRRNLIPHLPTSHEEASVLALQLTHFPNQGYSIGITSHHAALDGKSSTLFMKSWAHICSYLNTSPEEPLLFSLPKHLTPSFDRSVIRDPLGIGEIYAKSWTSFGGATNDRSLNVWDTLGGNQTDLVKGLFELTPLDIKKLKKLAESKFVVGDNKKKVRVTSFTVTCAYLLSCAVKAEQPNCERVHFVFNVDCRARLDPPIPETYFGNCVVALLASAKREELLGEEAFFKSVIGISEELNGLEGDVLNGADKWIPKIQSVVSETPRLFSVAGSPRFEVYGIDFGWGRPEKVDVASVNKTGAFSLSESRDHSGGIQIGLALTKNQMEAFSRVFAQGLESLES

>GmIF7MaT

MAETPTLRIHEVCPISPPQETPSTTIPFTFFDVLWLRLPPVERLFFYSFPNPTTTSSFFDTTILPNLKHSLSLTLHHFPPLAGTITWPLHTPLPLITYTPGNSIPFRIAESNADFNTLSSNLSEVNNHRRNLIPHLPTSHEEASVLALQLTHFPNQGYSIGITSHHAALDGKSSTLFMKSWAHICSYLNTSPEEPLLFSLPKHLTPSFDRSVIRDPLGIGEIYAKSWTSFGGATNDRSLNVWDTLGGNQTDLVKGLFELTPLDIKKLKKLAESKFVVGDNKKKVRVTSFTVTCAYLLSCAVKAEQPNCERVPFVFNVDCRARLDPPIPETYFGNCVVALLASAKREELLGEEAFFKSVIGISEELNGLEGDVLNGADKWIPKIQSVVSETPRLFSVAGSPRFEVYGIDFGWGRPEKVDVTSVDKTGAFSLSESRDHSGGIQIGLALTKNQMEAFSRVFAQGLESLES

>GmMT7

MAETPTLRIHEVCPISPPQETPSTTIPFTFFDVLWLRLPPVERLFFYSFPNPTTTSSFFDTTILPNLKHSLSLTLHHFPPLAGTITWPLHTPLPLITYTPGNSIPFRIAESNADFNTLSSNLSEVNNHRRNLIPHLPTSHEEASVLALQLTHFPNQGYSIGITSHHAALDGKSSTLFMKSWAHICSYLNTSPEEPLLFSLPKHLTPSFDRSVIRDPLGIGEIYAKSWTSFGGATNDRSLNVWDTLGGNQTDLVKGLFELTPLDIKKLKKLAESKFVVGDNKKKVRVTSFTVTCAYLLSCAVKAEQPNCERVPFVFNVDCRARLDPPIPETYFGNCVVALLASAKREELLGEEAFFKSVIGISEELNGLEGDVLNGADKWIPKIQSVVSETPRLFSVAGSPRFEVYGIDFGWGRPEKVDVTSVDKTGAFSLSESRDHSGGIQIGLALTKNQMEAFSRVFAQGLESLES

>MtMaT1

MASNNNSNIKVHDHFKVVPPSSTKTTSIPLTFFDIFWLRFHPVERVFFYTLPNSQSHPSFFFQTIVPNLKSSLSLTLQHFLPLAGNIVWPSDSSKPIIQFDPNDDGVSLIIAESDSDFNHVVENSPHEASLSRSFIPHLESSDSFASIMSLQITLFPNSGFSIGISTHHAVLDGKSSTMFVKAWAYLCKKAIERDESPTLLSEFEPSFNREVIKDPNGNNVMDLVSTLFPSEKGNDRSLKIFPFEPQLEDSVRATFKLKHEDLDKIKQRVLSTWEIFDTKESKPQTLSSFVITCAYSLVCVAKAIHGAHNDKEKFSFVFSVDCRARLEPTIPNNYLGNCVWAYFIDTQPLDFIKEDGVFLVAKSIYEKIKMINEKGFLEGEINDMFNKIISLSSEGFEFMGVAGSHRFGVYEIDFGWGRPEKVEIVSIDRGVTIGLAESKDSKGGIEVGLALNKPVMDIFSTLFLEGLSYNE

>MtMaT2

TTPLTYFDIFWLRFHPVERVFFYALPNSHSHPSFFFKKLVPILKSSLSLTLKDFLPLAGNIVWPLESQEPIIQYTPNDGVSLIIAESDVDFNHVIENSPHDASLSRCFVPHLESTNSFASIISVQITLFPESGFSIGISTHHAALDGKSSTMFIKAWAYLCNKTIETEELPTLLPELKPLLDREIIKDNGLGDKFTKNWTEIITMMFPNEKGNERSLKILPFEPKLEDYVRSTFKLTREDLNKIKQMVLSKWELLDTNELTSKPPTLSSFVLTCAYSLVCLAKAIHGVEKEKEKFGFAFTVDCRARLEPPLPNNYFGNCVWGHLVDTKPLDYINEDGVFLVAKCIHEKIKMINEKGVLDGVSDMFDKFASLASEKLEMMGVAGSNRFGVYEIDFGWGRPTKVEIVSADRGLTIALAESKDGKGGIEVGLVLNNHVMNLFRTLFVEGLCIN

>MtMaT3

MASLNKHIKIHEQCKVSPSSSSTQLSLPLTFFDYIWLRFHPVERIFFYTLPSSHSHPTFFFENLVPKLKSSLSLTLQHFLPLAGNIVWPSDSPKPFLQFNPNDDGVSLLLAQCDDDDVSFDKILEHNSPQEASLSRSFVPHLESSDSFASIISIQITLFPKNGFSIGISTHHAVLDGKSSTMFIKAWSSICKSLEEETQSLNLEPLLEPFLERELIEDPNDFENSFINTWNRISSHFDKSSVKSIKIMSSMFQPIIKDAVRETFELTREDLEKINKRVFSKWNNIEDGAQEKEQEQPKKLSTFVLTCAYVSVCIAKAIQQSESDKKQKFSIGFPVDCRSRLVPPIPKNYCGNCVSNHIVDTEPYDFTKEDGVVIVAKKIYGKTQEMDKGFLDGIETMMYKYMAMIGEGVKGIGVAGSTRFGVYEIDFGFGRPAKVEITSIDRGLTIGLTESKDLKGGVEIGLVLEKHVMDLFQAIFREGLCFD

>MtMaT4

MAFNKNNIKIHEHFKVVPSSSTQTTTIPFTFFDIFWIKFHPIERIFFYTLPNSQSHPSFFFQKLVPILRSSLSLTLQHFLPLAGKIVWPSESQQPKIQYTTNDGVSLLIAESDADFSHVIENSPHEASLSRSFLPHLESTDSSASIISIQITLFPKSGFSIGISTHHAGLDGKSSTMFMKAWAYLCHKIIETKEESPTLLPELEPLFDREVIKDPNELGVTFTNSWTEIISTIFPSEKGNEHSLNILHFEPKLEDSVRATFNLTREDLNKIKQMVLSKWEFYDTNESYSKPQTLSSFVLTCAYSLVCYAKAIHRADMEKEKFSFAFTVDCRARLEPPIPSNYFGNCVWGNFISTQPLDFIKKDGVFLVAKCIYEKIKMINENGVLEGAINDGYDKYNYLINEGFGVFGVSGSNRFGVYETDFGWGRPKKVEIVSVDRGLTIGFAESKDGNGGVEIGLVLNKHVMDLFSNLFLEGLCSN

>MtMaT5

MSTIPFILKVIEHCKITPPINTTQTVSSLPLTFFDIPWLLFSPSQPLFFYEFPHSISHFTTTIVPKLKQSLSLTLQHYFPFSGTFVPSLDLTEPQLEFTLNNSVSFTVAESNSDFEHLCSDYSRDVNEFHPLVPKLQQIFSFEVKEFPLLAIQITSFPNYGFSIGLAFHHVVADGRTFHNFIKTWSSYCSSSTFEDSSSLIKSLPLYDRSVIIDTNDLHEVFLKDWRKRRLVLNAKDSRESKVDSSNMTRATFLMSLTQMEKIKKIIVESCKEKKTSQPLHLSSYVLTSAFLWICLLKTQQEFINEKVVCEDVTHFGFIAGGITRLEYQVPKNYFGNCVGFGRVSLTKKDLLGEDGIVVAAKEIGSTIKKLDASIFGEGKKWILDWEMLHGSEEHVHVTWSPKLKLYELNFGWGRPKKIEEVSIDFTRGVSFVESRDFEGGIEIGLALPKSKMDIFTFFFKNGLEDLP

>MtMaT6

MGKPIGAEHKLIEQTLVFPATRTATTTYLPLTFLDLHFAGPIYAKRLFFYPFPHSTNHFCKTTLPSLKQSLSCALQHFFPLAGNLISPPPPQKPFIRCTEEDSVFFTIVESSSDFNHLSNKHHLKNLKENNHLAPILTHKTRVEDINDIENDTFTLPLLALQVSVFPNHGVCIGITYCHVMDGNSCNHFMKSWSFIHQGGDAAELKSLPCFDREVLKDPRGLEDLILRDYFFTRKSWKFRLIAQSQSTGEHEDSFKVIIAFGKEEIEGMKKWVLNQWKKNDNNEINVPKFISKFVVTSAFVWASMVEAMHKNDDGGDDDDEKDEYFCFTCDCRDRLGYSIPEGYFGNCVVSKSATMKRKDMKGIDSFVDVVKVIEKAVNEIKNEPLKKLEDWFELSKTMYMSGNQLFLHGSPKFNVYETNFGFGKPVKVEMVHAFKGVSLAESGDGEGGLEFGLVLKSEEFEYFSSLIQQGLEVFKY

>At5MaT

MVNFNSAVNILVVVRVSPPSSNSLTLPLTYFDLGWLKLHPVDRVLFYRVPELTRCSLITQIKSSLSVTLLHYLPLAGRLVWDSIDTKPSIVYSPNNDDAVYLTVAESNGDLSHFAGDKPRPAIDFHPLVPELSVSDESAGVLAVQITFFPNQGFCLGVTAHHAVLDGKTTAMFLKAWAHNCKQEQECGHIALPLPHDLIPSLDRTIVQDPTGLETKLLNRWISASNNKPSLKLFPSKIIGSDILRVTYRLTQEDIKKLRERVETESHAKQLRLSTFVITYAYVITCMVKMRGGDPTRFVCVGFASDFRSRLNPLLPSTFFGNCIVGSGDFDREGKGFISAVKSFTGWVNGLCPENIEKNMLLPFEAFKRMEPGRQMISVAGSNRLGIYGSNFGWGKPVKVEIVTIEKDGSVSLSESGDGS

DGVEIGICLKKDDVERFCSLFSRGLK

>NtMAT1

XASVIEQCQVVPSPGSATELTLPLTYFDHVWLAFHRXRRILFYKLPISRPDFVQTIIPTLKDSLSLTLKYYLPLAGNVACPQDWSGYPELRYVTGNSVSVIFSESDXDFNYLIGYHPRNTKDFYHFVPQLAEPKDAPGVQLAPVLAIQVTLFPNHGISIGFTNHHVAGDGATIVKFVRAWALLNKFGGDEQFLANEFIPFYDRSVIKDPNGVGXSIWNEXKKYKHXXKXSDVVTPPDKVRGTFIITRHDIGKLKNLVLTRRPKLTHVTSFTVTCAYVWTCIIKSEAATGEEIDENGXEFFGCAADCRAQFNPPLPPSYFGNALVGYVARTRQVDLAGKEGFTIAVELIGEAIRKRXKDEEWILSGSWFKEYDKVDAKRSLSVAGSPKLDLYAADFGWGRPEKLEFVSIDNDDGISXSLSKSKDSDGDLEIGLSLSKTRXNAFAAXFTHGISFL

>Dv3MAT

MDNIPNLTILEHSRISPPPSTIGHRSLPLTFFDIAWLLFPPVHHLYFYHFPYSKSHFTETVIPNLKHSLSITLQHYFPFVGKLIVYPNPHDSTRKPEIRHVEGDSVALTFAETTLDFNDLSANHPRKCENFYPLVPPLGNAVKESDYVTLPVFSVQVTYFPNSGISIGLTNHHSLSDANTRFGFLKAWASVCETGEDQPFLKNGSPPVFDRVVVNPQLYENRLNQTRLGTFYQAPSLVGSSSDRVRATFVLARTHISGLKKQVLTQLPMLEYTSSFTVTCGYIWSCIVKSLVNMGEKKGEDELEQFIVSVGCRSRLDPPLPENYFGNCSAPCIVTIKNGVLKGENGFVMAAKLIGEGISKMVNKKGGILEYADRWYDGFKIPARKMGISGTPKLNFYDIDFGWGKAMKYEVVSIDYSASV

SLSACKESAQDFEIGVCFPSMQMEAFGKIFNDGLESAIAS

>Dm3MAT2

MASLPILTVLEQSQVSPPPDTLGDKSLQLTFFDFFWLRSPPINNLFFYELPITRSQFTETVVPNIKHSLSITLKHFYPFVGKLVVYPAPTKKPEICYVEGDSVAVTFAECNLDLNELTGNHPRNCDKFYDLVPILGESTRLSDCIKIPLFSVQVTLFPNQGIAIGITNHHCLGDASTRFCFLKAWTSIARSGNNDESFLANGTRPLYDRIIKYPMLDEAYLKRAKVESFNEDYVTQSLAGPSDKLRATFILTRAVINQLKDRVLAQLPTLEYVSSFTVACAYIWSCIAKSRNDKLQLFGFPIDRRARMKPPIPTAYFGNCVGGCAAIAKTNLLIGKEGFITAAKLIGENLHKTLTDYKDGVLKDDMESFNDLVSEGMPTTMTWVSGTPKLRFYDMDFGWGKPKKLETVSIDHNGAISINSCKESNEDLEIGVCISATQMEDFVHIFDDGLKAYL

>Dm3MAT1

MASLPILTVLEQSQVSPPPDTLGDKSLQLTFFDFFWLRSPPINNLFFYELPITRSQFTETVVPNIKHSLSITLKHFYPFVGKLVVYPAPTKKPEICYVEGDSVAVTFAECNLDLNELTGNHPRNCDKFYDLVPILGESTRLSDCIKIPLFSVQVTLFPNQGIAIGITNHHCLGDASTRFCFLKAWTSIARSGNNDESFLANGTRPLYDRIIKYPMLDEAYLKRAKVESFNEDYVTQSLAGPSDKLRATFILTRAVINQLKDRVLAQLPTLEYVSSFTVACAYIWSCIAKSRNDKLQLFGFPIDRRARMKPPIPTAYFGNCVGGCAAIAKTNLLIGKEGFITAAKLIGENLHKTLTDYKDGVLKDDMESFNDLVSEGMPTTMTWVSGTPKLRFYDMDFGWGKPKKLETVSIDHNGAISINSCKESNEDLEIGVCISATQMEDFVHIFDDGLKAYL

>Vh3MAT1

MATTTRKVTVRERCGIAPPADAGEAVEQRLPITYFDTIWLYFHPIQRLLFYQYPCSKTHFVEHLVPNLKKSLKQTLRHYRPLAGKLIRPVDSGMPELRYSPGDSVSVTFAETNGDFDFNHLTGNHVRDSDEFYSFAPDLPEPVTEPDPGFTVVPLFAIQVTLFPEVGICMGFTNHHAVGDASSIVGFIKSWSSVAKSGGDEILAQKNSLPFYDRSVIKDPSGRADILWNQMRTFQIGSDHSNFPTNRFRATFILRKHEIQHLKNLVAEKKPGLSHLSSFTVTTSYVWSCLAKASAESGEEVDETEPEYFGFAVDARHRMDPPAPAAYFGNCLAFVVVETTHGVLKGEDGFFTGVELVSEIISKKVNNKNELLRDAHEWVVKYGPIVGKRLVGVAGSPKFDLYDTDFGWGNPNKYESVSIDNDGSMSLCKSREFESGLEIGMSLPKKKMEAFVDAFRHGLKI

>Lp3MAT1

MNNSAKKVNVLEHSGVAPAAAADEVAEQRLPLTYFDVLWLYFHPIKRLLFYQHPCSATHFLQTIIPNLKNSLSQTLRRYPPLAGHLFYPLDSGFPELRYLPGDSVTVTFAESTEAFDFNYLTGDQARVADEFHHFVPDLPQHKIDSDSGFRIIPLLAIQVTLFPETGISVGFTNNHVAGDASSIVGFIKAWSSSSKLGHFAENLDRPLYDRSVIKDPSGKRANIFWNQMRAQIWPTRSNPPSNRVRKTFVLQSKDIKTLKDLVLAREANFSYLSSFTVTIAHVWACLAKSSAEAGEEVEDAEPEYFGVAVDARSRLDPTVPATYFGNCITLAAAESRRGEMKGKDGFFVAAELIGDVMSKKVNKKGELMRDADELLVKYAPLFSKRFYGVSGSPKFDLYDTDFGWGNPNKFEALSIDEESYSISLCKSREFEGGLEIGVSFPERKMDAFQAVFYDRLGIQT

>Ss5MaT1

MTTTTTILETCHIPPPPAANDLSIPLSFFDIKWLHYHPVRRLLFYHHPSSKSQFLHTIVPHLKQSLSLALTHYLPVAGNLLYPSNTEKFPQLRYAAGDSVPVTIAESNSDFESLTGNHTRDADQFYDLLPPIPPIEEESDWKLINIFAVQITLFPGEGICIGFSNHHCLGDARSIVGFISAWGEINGIGGYEGFLSNHSDSLSLPIFDRSFINDPNKIDAIFWKVLRNIPLKTASFPLPTNRVRSTFLLRRSDIEKLKTATKSPASSFVAAAAFVWSCMVKSGDKSDENAPELFIIPADARGRVDPPIPENYFGNCIVSSVAQVERGKLAAEDGFAVAAEAIGGEIEGKLKNRDEILRGAENWMSDIFKCFGMSVLGVSGSPKFDLLKADFGWGKARKLEVLSIDGENHSMSLCSSSDFNGGLEVGLSLPRERMAAFEEVFRASIMAASGPARRSPALVEPL

>Ss5MaT2

MNINQINTKLIKPITPTPQNLKNYHISFLDQHVVKKYIAVVLYYQSAPDNGRLEDSLAETLVHFYPLAGRYIKTDLTVDCSDQGAEFIEAEARGDVRVTDLIGKTDTIHLCPEQYFGLDEGVDDPLLSIQVTRFSCGGATIAVSVSHRVFDVSSLETFLSAWSSASKTGGGVAPVIPSFALASLLPNKDEKFGLDSNKCQGKEQKIAVKRLLFEKRALTRLTSERTSGVRAACAVIAKALIRLDRTTHGKSRDFVVFQPINMRGRTGVPSPKNACGNMSFGSFTRRVSAKEEVGIGELVGLIGDGVRRGIAEYTEILCPDRDGRDVIIHVRNKNIKEVFKSETFVVSFTDWSKFGFYEVDFGWGRPIWSGVGPQRPRGNQTIMMRSKEGDGIEAWVHLNEDDMDLFEQDVEIKLFLS

>Dm3MaT3

MASLPILTVLEQSQVSPPPDTLGDKSLQLTFFDFFWLRSPPINNLFFYELPITRSQFTETVVPNIKHSLSITLKHFYPFVGKLVVYPAPTKKPEICYVEGDSVAVTFAECNLDLNELTGNHPRNCDKFYDLVPILGESTRLSDCIKIPLFSVQVTLFPNQGIAIGITNHHCLGDASTRFCFLKAWTSIARSGNNDESFLANGTRPLYDRIIKYPMLDEAYLKRAKVESFNEDYVTQSLAGPSDKLRATFILTRAVINQLKDRVLAQLPTLEYVSSFTVACAYIWSCIAKSRNDKLQLFGFPIDRRARMKPPIPTAYFGNCVGGCAAIAKTNLLIGKEGFITAAKLIGENLHKTLTDYKDGVLKDDMESFNDLVSEGMPTTMTWVSGTPKLRFYDMDFGWGKPKKLETVSIDHNGAISINSCKESNEDLEIGVCISATQMEDFVHIFDDGLKAYL

**Supplementary Note_3:** List of soybean BAHD family genes most close to GmIMaT1 and 3 from soybean genome

**GmIMaT1 (Glyma.18G268200.1):** Glyma.18G029900.1, Glyma.18G103400.1, Glyma.18G103500.1, Glyma.18G104000.1, Glyma.18G104100.1, Glyma.18G113100.1, Glyma.18G258000.1, Glyma.18G268100.1, Glyma.18G268300.1, Glyma.18G268400.1, Glyma.18G268500.1, Glyma.18G268600.1, Glyma.18G271600.1

**GmIMaT3 (Glyma.13G056100.1):** Glyma.13G054000.1, Glyma.13G302300.1, Glyma.13G302700.1, Glyma.13G371000.1
